# Efficient, fast, simple, and eco-friendly methods for separation of toxic chromium(VI) ions based on ion exchangers and polymer materials impregnated with Cyphos IL 101, Cyphos IL 104, or D2EHPA

**DOI:** 10.1007/s11356-023-31648-5

**Published:** 2024-01-04

**Authors:** Katarzyna Witt, Małgorzata A. Kaczorowska, Daria Bożejewicz

**Affiliations:** Faculty of Chemical Technology and Engineering, Bydgoszcz University of Science and Technology, 3 Seminaryjna Street, 85326 Bydgoszcz, PL Poland

**Keywords:** Cr(VI) ions, Separation process, Sorption materials, Ionic liquids, D2EHPA

## Abstract

In this study, we present the results of the first comparison of the elimination of toxic Cr(VI) ions, which are hazardous contamination of the environment, from aqueous solutions using ion exchangers (IEs) and polymer materials (PMs) impregnated with D2EHPA or ionic liquids (Cyphos IL 101 and Cyphos IL 104). Sorption of Cr(VI) ions and desorption from the formulated sorption materials were carried out. In comparison, classical solvent extraction was accomplished. Fourier transform infrared-attenuated total reflectance spectroscopy (FTIR-ATR), scanning electron microscopy-energy dispersive spectroscopy (SEM-EDS), and atomic force microscopy (AFM) have been used for characterization of the structure of developed IEs and PMs. The highest efficiency of adsorption of Cr(VI) ions was obtained using PMs with ionic liquids (>82%). Desorption from these materials were also very efficient (>75%). On the contrary, the application of IEs allowed for obtaining the best results of both, sorption and desorption processes when using D2EHPA (75% and 72%, respectively). The application of PMs and IEs is part of the green chemistry, and the conducted elimination of chromium(VI) ions using developed materials allows for the conclusion that they can potentially be used on a larger scale, e.g., for the treatment of industrial wastewater rich in Cr(VI) ions.

**Supplementary Information:**

The online version contains supplementary material available at 10.1007/s11356-023-31648-5.

## Introduction

Chromium is a transition metal present in the environment, both as a result of natural processes (e.g., erosion of rocks leading to the release of chromium as a result of chromite oxidation) as well as human activity related to the development of various industrial branches (e.g., productions of steel, paints and textile dyes, ceramic glazes, and refractory bricks) (Tumolo et al. 2020; Hausladen et al. 2018; Zhang et al. 2022). The systematic increase in the use of chromium associated with anthropogenic activity, observed for several decades, makes the pollution of waters, soils, or plant crops by this element a serious problem (Oliveira 2012; Wang and Cui 2019; Gezahegn et al. 2021). However, the influence of chromium on living organisms depends on its oxidation state, the most stable and widespread forms existing in aqueous solutions are Cr(III) and Cr(VI), which is considered to be particularly dangerous (Sharma et al. 2022; Han et al. 2023). The risks associated with the presence of Cr(VI) ions are related to their long persistence in the environment, high toxicity to both plants and animals, and their carcinogenic, genotoxic, and mutagenic nature for humans (Gezahegn et al. 2021; Sharma et al. 2020). Cr(III) ions are considered less hazardous than Cr(VI), but due to increasingly stringent requirements for ecological production and emissions of hexavalent chromium, nowadays, the oxidation of Cr(III) is gradually replacing direct emissions as one of the major sources of Cr(VI) in soil and groundwater (Liang et al. 2021; Shenashen et al. 2013).

Because of the properties of Cr(VI), its wide use in various industries and the associated pollution of the environment, methods of efficient, economic, and safe removal of hexavalent chromium ions from water, sewage, or waste are systematically sought. Commonly used methods for heavy metal ions removal including Cr(VI) ions are chemical precipitation, adsorption, electrocoagulation, electrodialysis, ion exchange, and membrane separation (Qasem et al. 2021; Tabidi 2020). Chemical precipitation methods intended to eliminate chromium ions from wastewater are usually based on the use of simple and inexpensive chemical compounds (e.g., NaOH), but the processes themselves depend on many factors (e.g., pH, mixing speed and duration, and complexing agents), and additionally, it is necessary to separate and properly manage large amounts of generated sludges (Sharma et al. 2022; Giwa et al. 2019). Application of electrocoagulation and electrodialysis techniques (El-Taweel et al. 2015) for Cr(VI) ions removal is also related to formulation of concentrated wastes (Sharma et al. 2022; Chen et al. 2018). Adsorption methods are usually characterized by easy implementation; however, they also have disadvantages (e.g., they usually are not very effective for removing trace amounts of metal ions) and systematic research is being carried out on the possibility of using more effective modified adsorbents for Cr(VI) ions removal (e.g., carbon-based adsorbents, micellar modified adsorbents) (Chen et al. 2022a; Sarfraz et al. 2022; Badessa et al. 2020). The recent, increase in interest in implementing biosorption processes to remove hexavalent chromium ions has been observed, which is associated with, inter alia, the fact that these methods are considered as cost effective and environmentally friendly, although they are often time-consuming (Bayuo 2021; Łukomska et al. 2022). Because each of the used methods of removing Cr(VI) ions from aqueous medium has some drawbacks (e.g., insufficient efficiency, high cost, and generation of sludge), new methods are developed or well-known solutions are modified in order to increase the efficiency of the process, simplify the procedure and reduce costs.

The group of chemical compounds used in recent years to remove various metal ions from solutions or recover valuable metal ions from waste includes, among others, D2EHPA (di-(2-ethylhexyl)phosphoric acid) and ionic liquids such as Cyphos IL 101 (trihexyl(tetradecyl)phosphonium chloride) and Cyphos IL 104 (trihexyl(tetradecyl)phosphonium bis(2,4,4-trimethylpentyl)phosphinate). For environmental and economic reasons, the use of well-known, commercially available compounds for the removal of metals, especially those as hazardous as Cr(VI), or recovery of precious metals, natural deposits of which are systematically declining, is of particular interest, and much research is being done in this area. For example, Cyphos IL 101 has been successfully applied in solvent extraction for the recovery of nickel and cadmium from the “black mass” of spent Ni-Cd batteries (Mahandra et al. 2022), gold from thiosulfate leachate of sulfidic gold ore (Paiva et al. 2022), and platinum-group metals from spent auto-catalysts (Chen et al. 2022b). Cyphos IL 104 proved to be an efficient extractant in ionic liquid extraction of rare earth elements (REEs) (Lu et al. 2022; Wiecka et al. 2022) as well as platinum group metals (Tang et al. 2022) from different types of leachates obtaining from waste (e.g., spent neodymium magnets, automotive converters). D2EHPA has been used, among others, in solvent extraction intended for vanadium removal from aqueous leachate of stone coal after low-temperature sulfation roasting and of indium recovery from acidic leaching solutions (Grigorieva et al. 2022; Bensaadi et al. 2022). All three compounds have been effectively used as carriers in polymer inclusion membranes intended for chromium(VI) ions removal from aqueous solutions (Bahrami et al. 2020; Guo et al. 2012). Ionic liquids are also increasingly utilized in impregnated resins to remove various metal ions (Sakshi et al. 2020). For example, Yang et al. (2012) used solvent impregnated resin (XAD–7) with Cyphos IL 104 for Cr(VI) removal and found that ion liquid existed in the inner XAD-7 resin and that the process efficiency was optimal when the pH was in the range of 0–2. Recently, Verma et al. (2022) reported that anion exchange resin with phenol–formaldehyde matrix can be successfully applied for Cr(VI) ions removal from aqueous solution and that in addition to ion exchange reactions, redox reactions also were occurring inside the resin (reduction of Cr(VI) to Cr(III)). Due to the properties of Cyphos IL 101, Cyphos IL 104, and D2EHPA, these compounds can potentially be used on a larger scale in the future for the removal of Cr(VI) ions, e.g., from industrial wastewater. However, before that happens, it is necessary to determine which of the separation methods is the most efficient and beneficial from the environmental and economic point of view.

In this manuscript, we present for the first time a comparison of the efficiency of the separation processes of toxic chromium(VI) ions using ion exchangers (IEs) and polymer materials (PMs) impregnated with ionic liquids (Cyphos IL 101 and Cyphos IL 104) and D2EHPA. The obtained results clearly show that in principle, analyzed materials can be successfully applied for removal of toxic hexavalent chromium ions from aqueous solutions, but their effectiveness strongly depends on the type of sorption materials and methods in which they are used.

## Experimental

### Materials

All the reagents used in this work were of analytical grade purity and were used without further purification. The reagents: stock nitric solution of chromium(VI) ions with the concentration of 1000 mg/L and pH  about 1, Cyphos IL 101, Cyphos IL 104, and D2EHPA, were purchased from Sigma Aldrich. The other compounds used in experiments, such as nitric acid, chloroform, and methanol were bought from Avantor (Gliwice, Poland). The poly(vinyl chloride) (PVC) in suspension with an average molecular weight of 72,000 was obtained from the Anwil Company (Poland). The bis(2-ethylhexyl)adipate (ADO) and tetrahydrofuran (both of analytical grade) were purchased from Avantor (Gliwice, Poland). Nitric acid was standardized against anhydrous sodium carbonate. The ion exchanger 001X8 H used in this study is made of polystyrene and has a gel structure and a strongly acidic character. The cation exchanger has the hydrogen form and functional groups R-SO_3_^−^. The particle size range of the cation exchanger is equal to 0.3–1.2 mm, the capacity is 1.2 val/min, the moisture retention is 50–55%, and maximum thermal resistance is 150°C.

### Liquid–liquid extraction

The chromium(VI) ions were separated from the aqueous solution using liquid–liquid extraction. The measurements were run at 25±0.2 °C, at a fixed ionic strength (0.5 mol/L) maintained in the aqueous phase with KNO_3_. The organic phase contained one of the extractants (Cyphos IL 101, Cyphos IL 104, or D2EHPA) diluted in chloroform. The aqueous phase contained Cr(VI) ions. The appropriate volumes of both phases (aqueous and organic phase) were mixed in order to obtain a molar ratio of metal ion to ligand in each sample equal to 1:2. Equilibrium was established after approximately 5 min through visual observation. It was checked if any changes in phase volumes had occurred, then the phases were separated and to control the process the pH of the aqueous phase was measured. The concentration of metal ions in aqueous phases was determined with the use of atomic absorption spectroscopy (Thermo Scientific ICE 3000). The pH-meter (Mettler Toledo, Greifensee, Switzerland) utilized in the performed experiments was calibrated using commercial technical buffer solutions (Mettler Toledo, Greifensee, Switzerland) with a pH of 2.00, 4.01, 7.00, and 10.00.

The liquid–liquid extraction of chromium(VI) ions from aqueous nitric solutions was described and calculated using extraction efficiency (*%E*) (Witt et al. 2021).

### The sorption materials obtaining

The ion exchangers (IEs) and polymer materials (PMs) impregnated with appropriate ion carriers were used as sorption materials. The PMs were prepared by dissolving the poly(vinyl chloride), appropriate ion carrier (Cyphos IL 101 or Cyphos IL 104 or D2EHPA) and plasticizer (ADO) in 10 mL of tetrahydrofuran. Each solution prepared in this way was poured into ANUMBRA self-leveling Petri dish with a diameter of 4.5 cm. The dish was covered with filter paper to allow the THF to slowly evaporate over 1 day. After complete evaporation of solvent, the membrane was carefully separated from the glass plate and conditioned in distilled water for 24 h. The obtained PMs contained a 60 wt.% of poly(vinyl chloride), 20 wt.% of suitable ion carrier and a 20 wt.% of plasticizer.

The impregnation of the ion exchangers was carried out as follows:

1. First, the ion exchanger 001X8 H had to be prepared and purified. The 200 g of ion exchanger was weighed and then washed with organic solvent (acetone) and demineralized water to remove residues and pollutants from post-production processes. The ion exchanger prepared in this way was dried in natural air circulation at temperature of 60°C using the SUP-30G Wamed laboratory dryer to dry mass.
2. Two grams of an appropriate ion carrier (Cyphos IL 101, Cyphos IL 104, or D2EHPA) was added to 5 g of ion exchangers and stirred on a magnetic stirrer for 4 h.
3. Then, the obtained ion exchangers were filtered off from the carriers’ residues, washed abundantly with distilled water, and dried again.

**Fig. 1:**
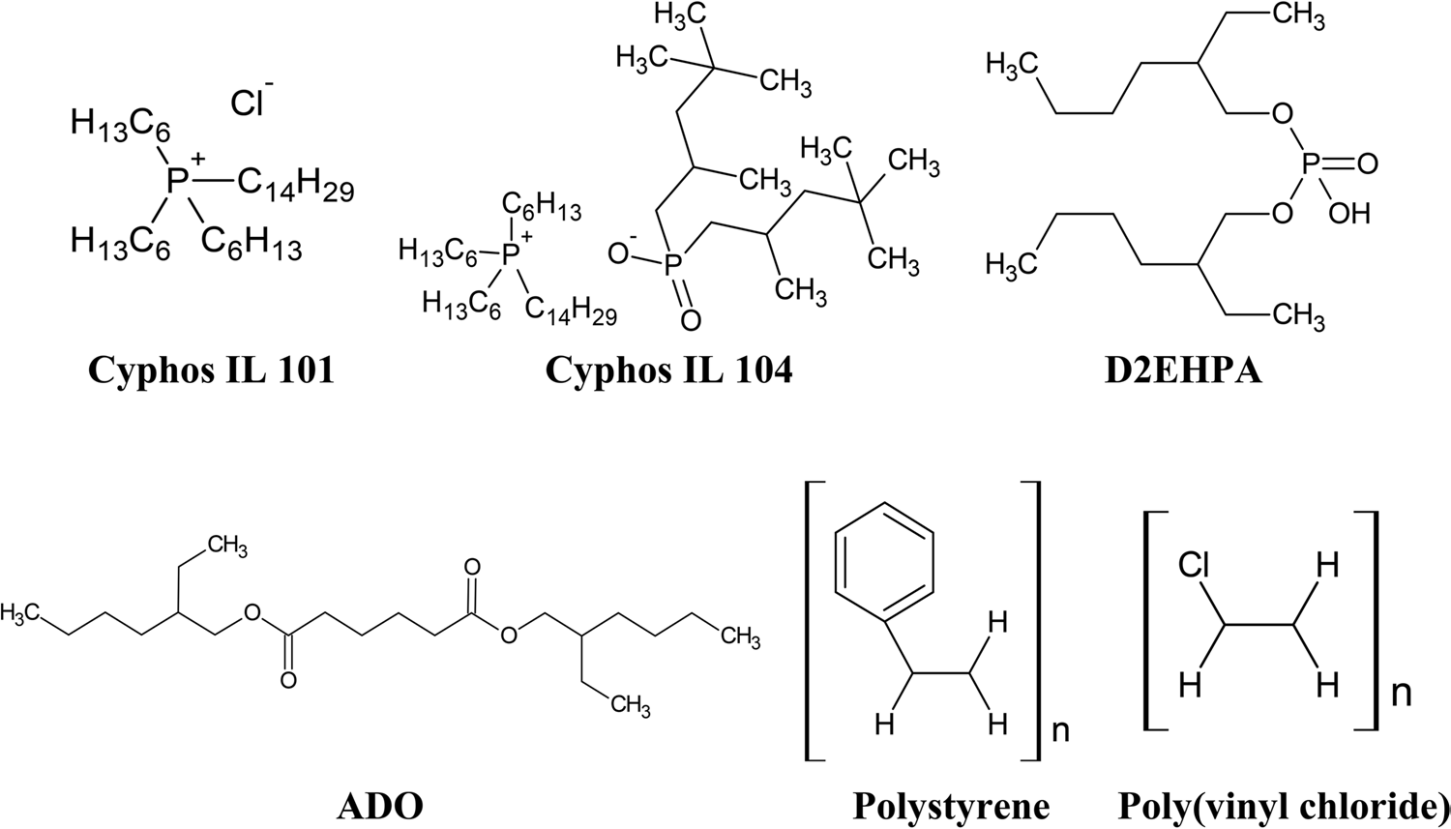
The structures of compounds used to obtain the ion exchangers and polymer materials, where: Cyphos IL 101—trihexyl(tetradecyl)phosphonium chloride, Cyphos IL 104—trihexyl(tetradecyl)phosphonium bis(2,4,4-trimethylpentyl)phosphinate, D2EHPA—di-(2-ethylhexyl)phosphoric acid, ADO—bis(2-ethylhexyl)adipate

Figure 1 shows the structures of compounds used to obtain the ion exchangers and polymer materials.

### Characterization of sorption materials

The structure of the obtained ion exchangers (from IE-0 to IE-3) and polymer materials (from PM-0 to PM-3) before and after sorption processes was investigated by Fourier transform infrared—attenuated total reflectance (FTIR-ATR) spectroscopy, scanning electron microscopy—energy-dispersive spectroscopy (SEM-EDS), and atomic force microscopy (AFM). The FTIR spectra were obtained on a Bruker ALPHA spectrometer at a wavenumber range of 350–2000 cm^−1^. The SEM-EDS were obtained on a scanning electron microscope with focus ion beam (FEI Helios Nanolab 660). The AFM were made using atomic force microscope (Agilent 5500).

The thickness of sorption materials was determined after measuring the test samples with the manual precision thickness gauge Panametrics® Magna-Mike® 8500 (San Diego, CA, USA).

### Sorption process

The chromium(VI) ions sorption and desorption processes were carried out. As a feed phase, a standard solution of chromium(VI) ions in nitric acid with a concentration of 1000 mg/L was used. The appropriate volume of that aqueous solution was from 1.4 mL (IE-2) to 16.0 mL (PM-3) (Table 1). The volumes were different in each case, because they were calculated taking into account the molar ratio of the ion carrier to metal ion in the solution. This ratio in each case was 2:1. The polymer materials and ion exchanges were immersed in beakers with a prepared volume of aqueous solutions of Cr(VI) ions. After 10, 20, 40, 60, 120, 180, and 1440 min from the beginning of the sorption process, the small samples of the aqueous solutions were taken. The concentration of metal ions in aqueous phases was determined with the use of atomic absorption spectroscopy (Thermo Scientific ICE 3000).

**Table 1:** The volumes of aqueous solutions containing chromium(VI) ions used for sorption processes

Symbol of sorption material	Ion carrier	Volume of feed phase, mL
IE-0	Without ion carrier	3.20
IE-1	Cyphos IL 101	2.00
IE-2	Cyphos IL 104	1.40
IE-3	D2EHPA	3.20
PM-0	Without ion carrier	16.00
PM-1	Cyphos IL 101	9.98
PM-2	Cyphos IL 104	7.41
PM-3	D2EHPA	16.00

The given values with a tolerance volume ±0.01 mL

The sorption capacity (*q*_*t*_) of chromium(VI) ions from aqueous solutions on investigated sorption materials and the efficiency of sorption (%*R*_ads_) and desorption (%R_des_) processes were determined using following Eqs. (1–3):

1 $${q}_t=\left(\frac{c^i-{c}^t}{m}\right)\cdot V$$

2 $$\%{R}_{ads}=\left(\frac{c^i-{c}^t}{c^i}\right)\cdot 100\%$$

3 $$\%{R}_{des}=\left(\frac{c^i}{c^a}\right)\cdot 100\%$$

where *q*_*t*_ is the sorption capacity (mg/g), *V* is the volume of the solution (L), *m* is the mass of the used sorption material (g), and *c*^*i*^ and *c*^*t*^ are the analytical concentration of metal ions in the solution at the beginning and after a determined period of the sorption process (mol/L), respectively, and *c*^*a*^ refers to the initially sorbed concentration of metals during the desorption process.

### Desorption process

After each sorption process, the sorption materials were immersed in 10 mL of 5 mol/L HNO_3_ solution for 24 h to desorb chromium(VI) ions. After that, the sorption material was removed from the solution, in which the concentration of Cr(VI) ions was determined by AAS method. A desorption process was conducted to assess the degree of recovery of chromium ions from the sorption materials to the concentrated aqueous solution.

## Results

### Liquid–liquid extraction

The results of extraction efficiency (*%E*) are presented below (Table 2).

**Table 2:** The results obtained after 24 h of liquid–liquid extraction processes with different extractants

Extractant	%E
Cyphos IL 101	46.72
Cyphos IL 104	64.15
D2EHPA	5.84

The given values with a tolerance %E ± 0.01%

In given conditions, separation of chromium(VI) ions with tested extractants (Cyphos IL 101, Cyphos IL104, and D2EHPA) occurs. The *%E* parameter for those three processes has divergent values. The best value of *%E* reached merely 64.15% and the worst only 5.84%. Chromium ions can be removed the most efficiently from aqueous solution during liquid–liquid extraction with Cyphos IL 104, and with the least efficiency in case of D2EHPA. These results show that liquid–liquid extraction of chromium(VI) ions in chosen conditions and with investigated extractants are not very efficient.

**Fig. 2:**
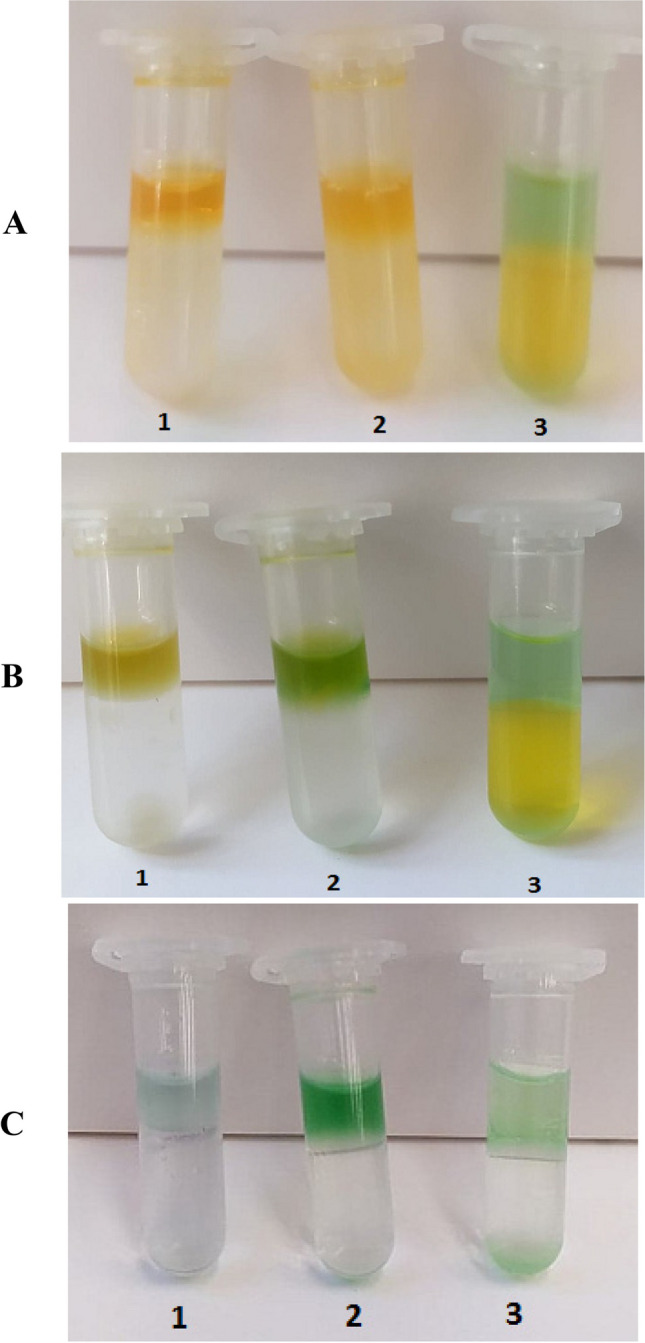
The processes of liquid-liquid extraction of chromium(VI) ions with tested extractants (1. Cyphos IL 101, 2. Cyphos IL104, and 3. D2EHPA) after: **A** 20 min., **B** 180 min., and **C** 48 h

The images (Fig. 2) present the conducted liquid-liquid extraction processes after different spans of time. The differences between colors of organic and aqueous phases are clearly visible in the three consecutive photos presented in Fig. 2A–C. After 180 min. and finally after 48 h of the performed extraction processes the orange color of created in organic phases complexes have changed into green and the color of the aqueous solutions have changed to transparent. This fact proves the transfer of chromium(VI) ions into the organic phase. The green color is related to the change in oxidation state of chromium ions (from Cr(VI) to Cr(III)). Similar behavior has been observed before by Verma et al. (2022). The pictures show that equilibria of complexation reactions, which were taking place between the chromium(VI) ions and the extractants, were established over a long period of time. The unsatisfactory results obtained for chromium(VI) ions separation after 24 h of establishing the extraction equilibria probably can be explained by this fact.

### Sorption process

**Fig. 3:**
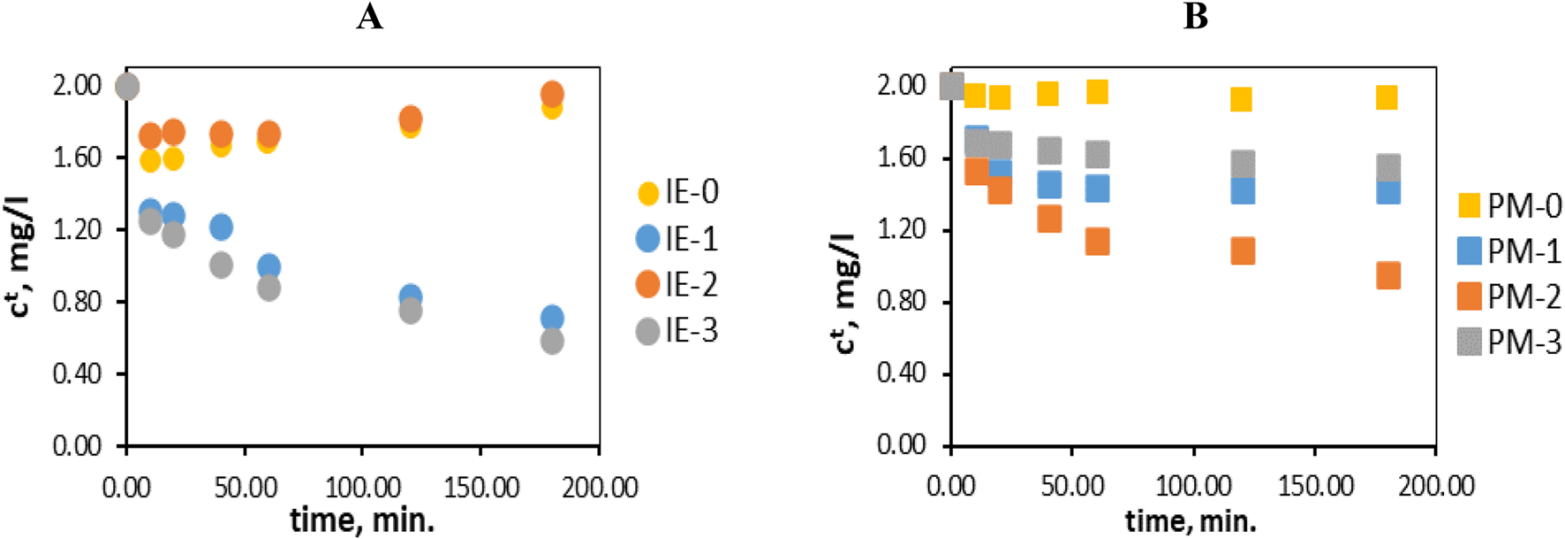
Changes in the concentration of the chromium(VI) ions in the solution over time during the sorption on impregnated: **A** ion exchangers and **B** polymer materials. The values given with a tolerance *c*^*t*^ carry ± 0.01

The kinetics of adsorption of chromium(VI) ions using such materials as IEs and PMs impregnated with ionic liquids (Cyphos IL 101 and Cyphos IL 104) and D2EHPA were investigated. Changes over time in the concentration of the chromium(VI) ions in the solution are shown in Fig. 3.

Figure 3A shows that concentration of chromium(VI) ions in the solution decrease only in the case of the application of IE-1 and IE-3, containing Cyphos IL 101 or D2EHPA, respectively. The IE-0 (without ion carrier) sorbed the small amount of metal ions and the same situation takes place unexpectedly with use of IE-2 (with Cyphos IL 104). This fact can be related to improper or incomplete impregnation of Cyphos IL 104 into the cation exchanger or to the properties of this ionic liquid, which under experimental conditions (such as the impregnation of ion exchanger) causes it to not sorb chromium ions well. As shown in Fig. 3B. concentration of chromium(VI) ions decrease for all used polymer materials with all types of ion carriers (PM-1 to PM-3). The PM-0 without carrier does not adsorb chromium(VI) ions.

**Fig. 4:**
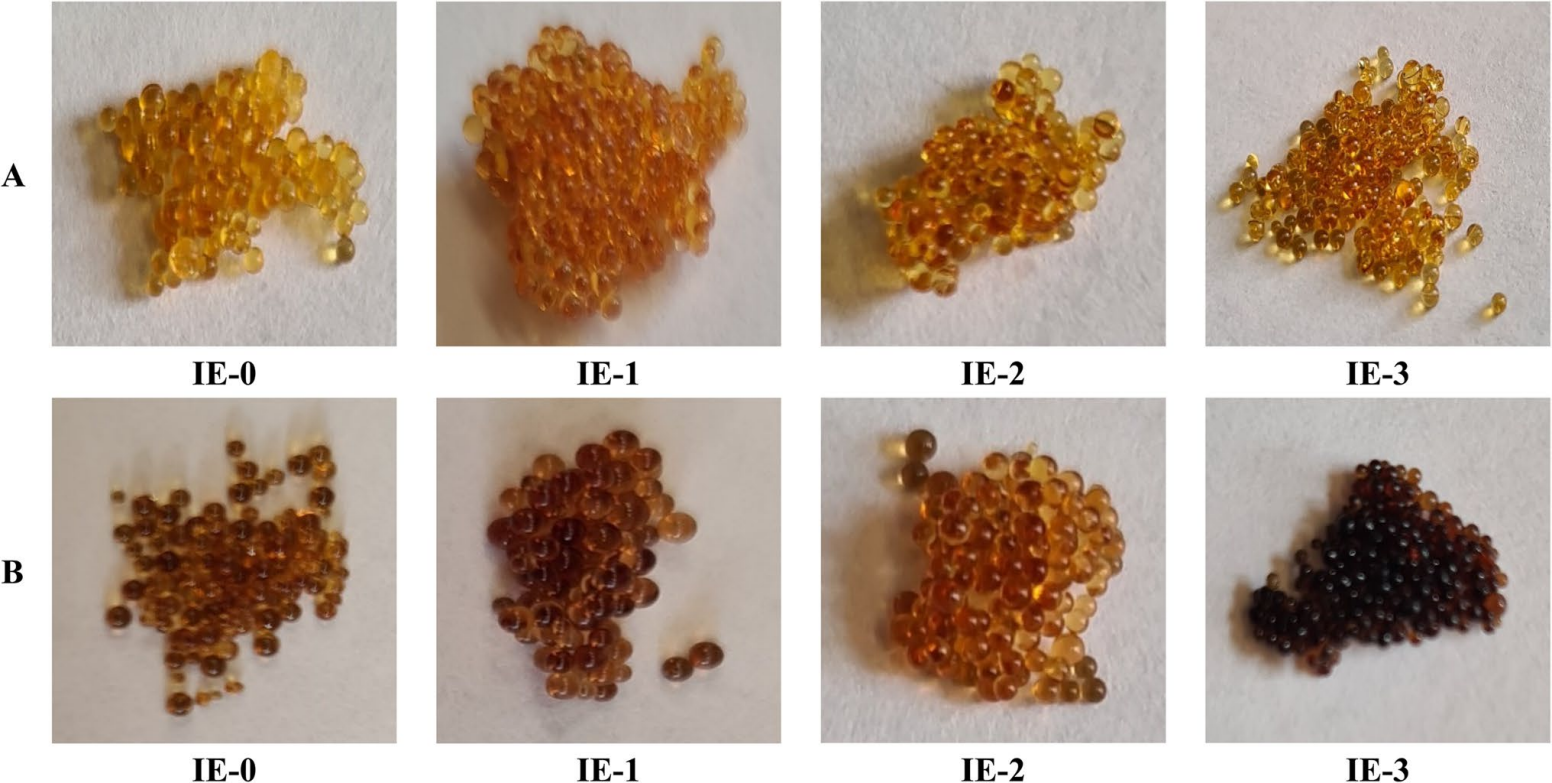
The images of ion exchangers before (**A**) and after (**B**) sorption processes

Figure 4 shows the ion exchangers (from IE-0 to IE-3) before (A) and after (B) sorption processes.

**Fig. 5:**
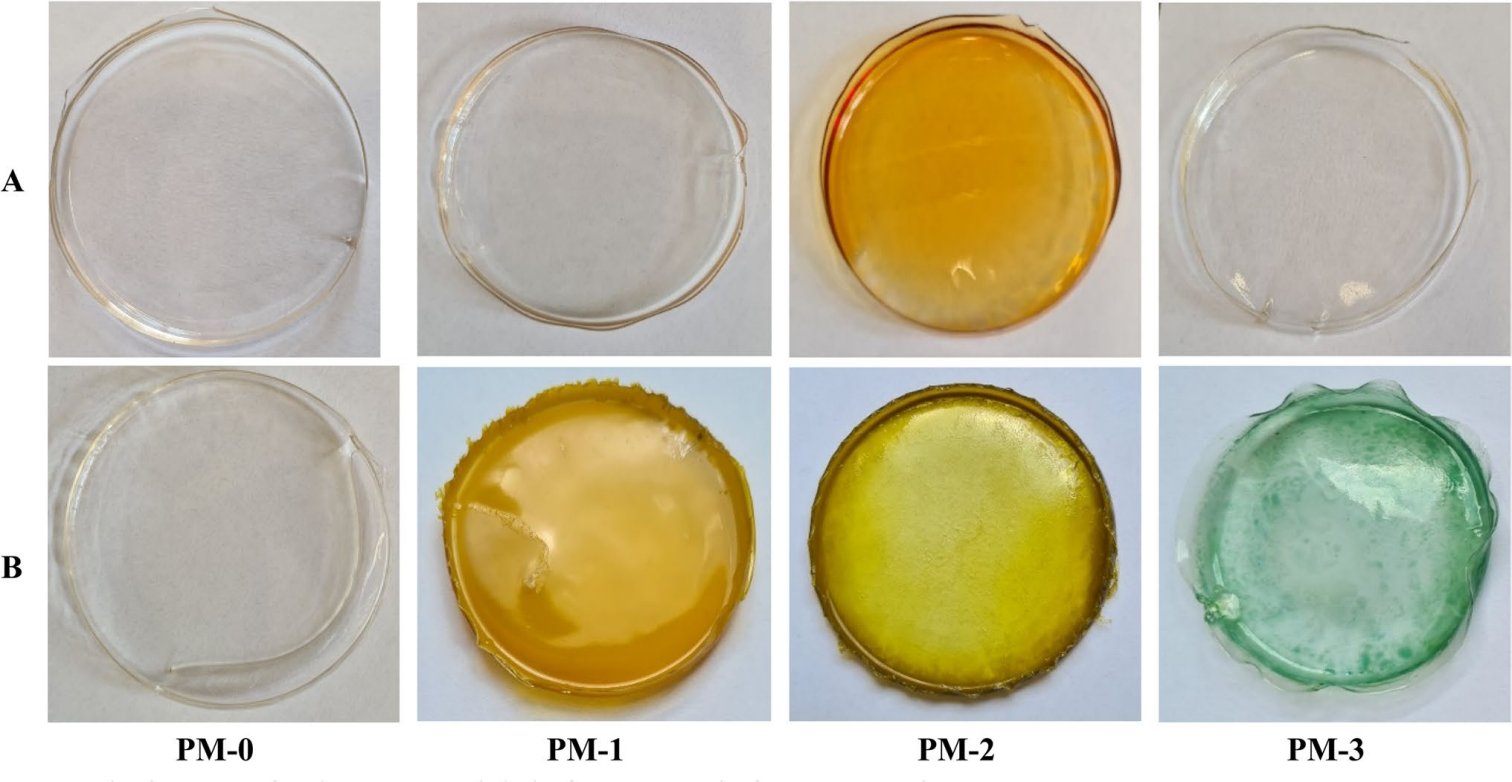
The images of polymer materials before (**A**) and after (**B**) sorption processes

In turn, Fig. 5 shows the polymer materials (from PM-0 to PM-3) before (A) and after (B) sorption processes.

The changes in the appearance of tested ion exchangers and polymer materials are clearly visible after the sorption processes. The samples have a darkened color, which is caused by the binding of chromium(VI) ions from the solution. The bright color of IE-0, IE-2, and PM-0 confirm the results present in Fig. 3A that only a small amount of metal ions were sorbed by these agents. All of the surfaces of PMs (except PM-0) were covered by chromium ions. In case of PM-3 the green color is visible, which also occurs during the extraction process with D2EHPA (change in oxidation state of chromium ions).

The sorption capacities (*q*_*t*_) of investigated IEs and PMs were determined according to Eq. (1) and summarized in Table 3.

**Table 3:** Comparison of the sorption capacities of the tested sorption materials: IEs and PMs

*t*, [min]	IE-0	IE-1	IE-2	IE-3	PM-0	PM-1	PM-2	PM-3
	*q*_*t*_, mg/g							
**10**	12.76	13.15	3.65	22.90	0.86	3.14	3.89	7.59
**20**	12.41	13.45	3.38	25.14	1.02	5.00	4.62	7.89
**40**	10.10	14.54	3.38	30.06	0.67	5.55	5.74	8.37
**60**	9.42	18.37	3.41	33.74	0.50	5.67	6.60	8.72
**120**	6.73	21.31	2.20	37.23	1.15	5.61	6.80	9.95
**180**	3.69	23.22	0.45	41.99	0.97	5.55	7.55	10.35
**1440**	**0.16**	**14.52**	**0.39**	**44.61**	**0.48**	**15.49**	**10.29**	**16.18**

The given values with a tolerance *qt* ± 0.01 mg/g

The sorption capacities of the tested ion exchangers and polymer materials in most cases (IE-3, PM-1, PM-2, PM-3) increase from the beginning of the process, with the highest values being reached after 24 h (Table 3). The IE-1 binds the most chromium(VI) ions up until 180 min (23.22 mg/g), after which time the sorption capacity decreases. In the cases of IE-0 and IE-2, during the process the obtained *q*_*t*_ slowly decreased, whilst the *q*_*t*_ of PM-0 remains within the range of 0 to 1 mg/g. The highest sorption capacity after 24 hours of sorption were found for ion exchanger IE-3 with D2EHPA (44.61 mg/g) and the lowest for ion exchanger IE-2 with Cyphos IL 104 (0.39 mg/g). The sorption capacity of the multiwall carbon nanotubes impregnated with D2EHPA applied to remove chromium ions was 0.96 mg/g (Vellaichamy and Palanivelu 2010).

**Fig. 6:**
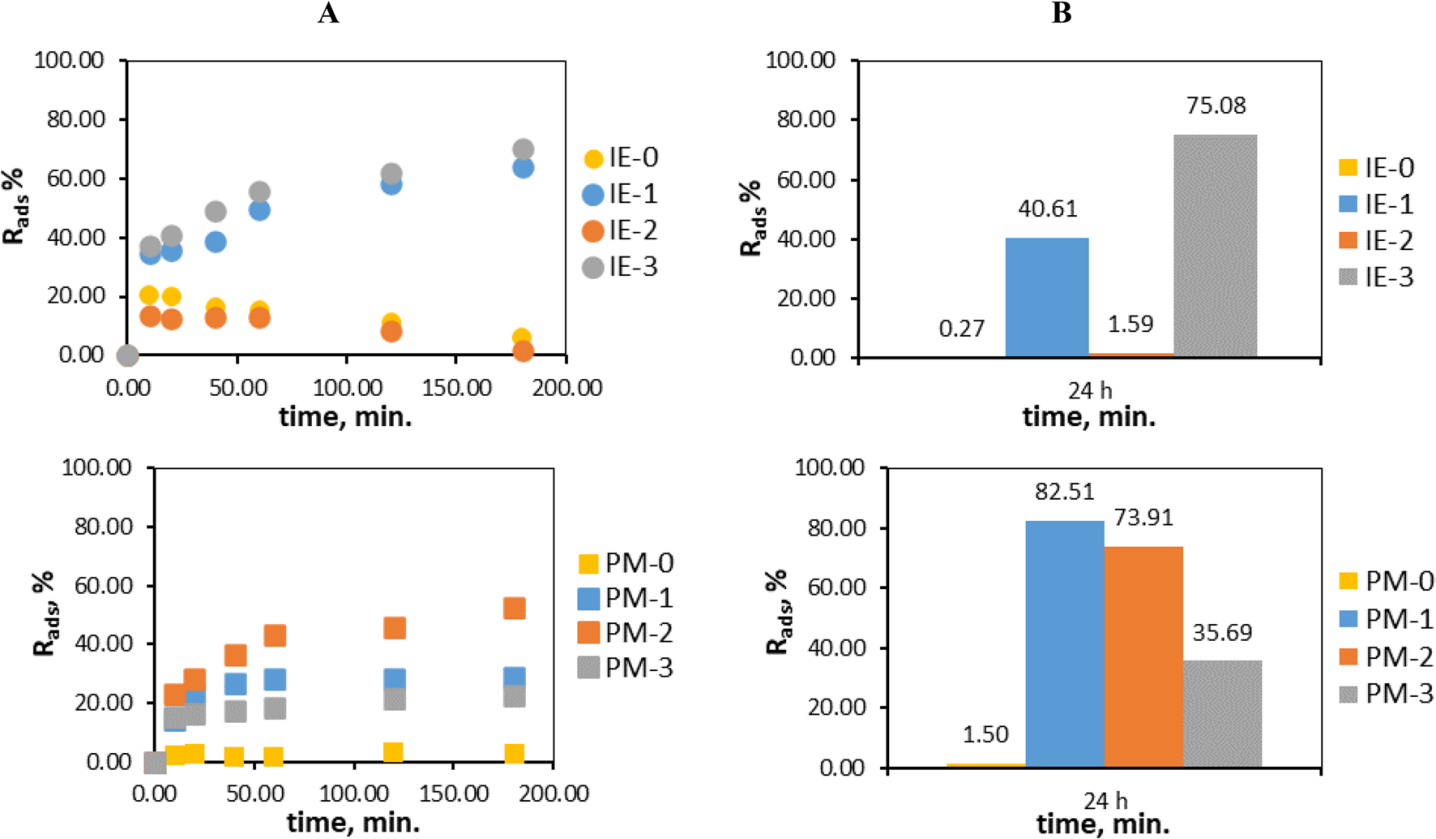
Changes in the efficiency of sorption (*R*_ads_) of the chromium(VI) ions from the aqueous solution on tested IEs and PMs: **A** for a period of 0–180 min. and **B** after 24 h

The efficiency of sorption (%R_ads_) processes conducted on the tested materials in aqueous solution were calculated and presented in Fig. 6.

Figure 6 shows the results (*R*_ads_) obtained during the sorption processes conducted for a period of 0–180 min. and after 24 h.

It was found that efficiency of PMs increases over time and achieves the highest results after 24 h of sorption, the same process takes place on IE-1 and IE-3 ion exchangers, while in the case of IE-0 and IE-2 ion exchangers *R*_ads_ decreases over time. As was mentioned above, this fact can be related to improper or incomplete impregnation of Cyphos IL 104 into the cation exchanger. The low sorption of metal ions on the non-impregnated cation exchanger proves the low affinity of the raw material to chromium(VI) ions and confirms the legitimacy of its impregnation.

The most effective polymer materials were those impregnated with Cyphos IL 101 and Cyphos IL 104 (PM-1 and PM-2), their *R*_ads_ reached the highest values after 24 h and were equal 82.51% and 73.91% respectively. However, the impregnation of the polymer material with D2EHPA acid did not bring satisfactory results (*R*_ads_ for PM-3 is equal to 35.69%), in contrast to the IE-3 ion exchanger impregnated with the same chemical compound (*R*_ads_, 75.08%). The obtained results are reflected in the literature data, for example, when using polymer inclusion membranes (PIM) containing D2EHPA as an ion carrier, the recovery of chromium(VI) ions was found at the level of approx. 43% (Bahrami et al. 2020). On the other hand, the use of Cyphos IL 104 as a carrier for metal ions in PIM allowed the recovery of 99.5% of Cr(VI) ions (Guo et al. 2011), while Cyphos IL 101 used as a carrier allowed the recovery of about 86% to about 98% of chromium(VI) ions (Wang et al. 2020). To sum up, the binding efficiency of obtained PMs for chromium(VI) ions can be ranked in the following order: PM-1 (82.51%) > PM-2 (73.91%) > PM-3 (35.69%) > PM-0 (1.50%). However, in the case of impregnated ion exchangers, their effectiveness decreases according to the following series: IE-3 (75.08%) > IE-1 (40.61%) > IE-2 (1.59%) > IE-0 (0.27%). It was found that chromium(VI) ions were best bound when the material was impregnated with D2EHPA acid (75.08%). Impregnation of the ion exchanger with Cyphos IL 104 was ineffective because this ion exchanger binds only 1.59% of chromium(VI) ions from the aqueous solution.

### Desorption process

After sorption processes, the desorption processes of chromium(VI) ions from the surface of the IEs and PMs impregnated by Cyphos IL 101, Cyphos IL 104, or D2EHPA were carried out. As a result of the desorption processes, the Cr(VI) ions adsorbed on an investigated sorption materials surfaces were transferred into the solution containing 5 mol/L HNO_3_. The results show that amounts of desorbed chromium(VI) ions from the surfaces of investigated sorption materials changed in the following order for IEs: IE-3 (72.99%) > IE-1 (40.61%) > IE-2 (0.00%) > IE-0 (0.00%), and for PMs: PM-1 (75.85%) > PM-2 (68.70%) > PM-3 (33.21%) > PM-0 (0.00%). The presented values show that almost all chromium(VI) ions previously bounded to the sorption materials passed into the acid solution. The desorption process allows the regeneration of the IEs and PMs and the reusing of them in further sorption processes.

### Characterization of investigated sorption materials (IEs and PMs)

#### The thickness

The mean value of the thickness of PMs and IEs resulted from 10 measurements, which were made for polymer materials at randomly selected points located on their surface, while for ion exchangers for randomly selected grains. The mean thicknesses for sorption materials were presented in the Table 4.

**Table 4:** The thickness of the tested sorption materials: IEs and PMs

IE-0	IE-1	IE-2	IE-3	PM-0	PM-1	PM-2	PM-3
Thickness, mm							
0.311	0.372	0.413	0.458	0.201	0.287	0.243	0.214

The given values with a tolerance thickness± 0.001

#### SEM-EDS

SEM-EDS method was used for the observation, analysis, and characterization of the surface of the tested sorption materials, both impregnated ion exchangers (from IE-0 to IE-3) and polymer materials (from PM-0 to PM-3), including mainly morphology and elemental composition.

**Fig. 7:**
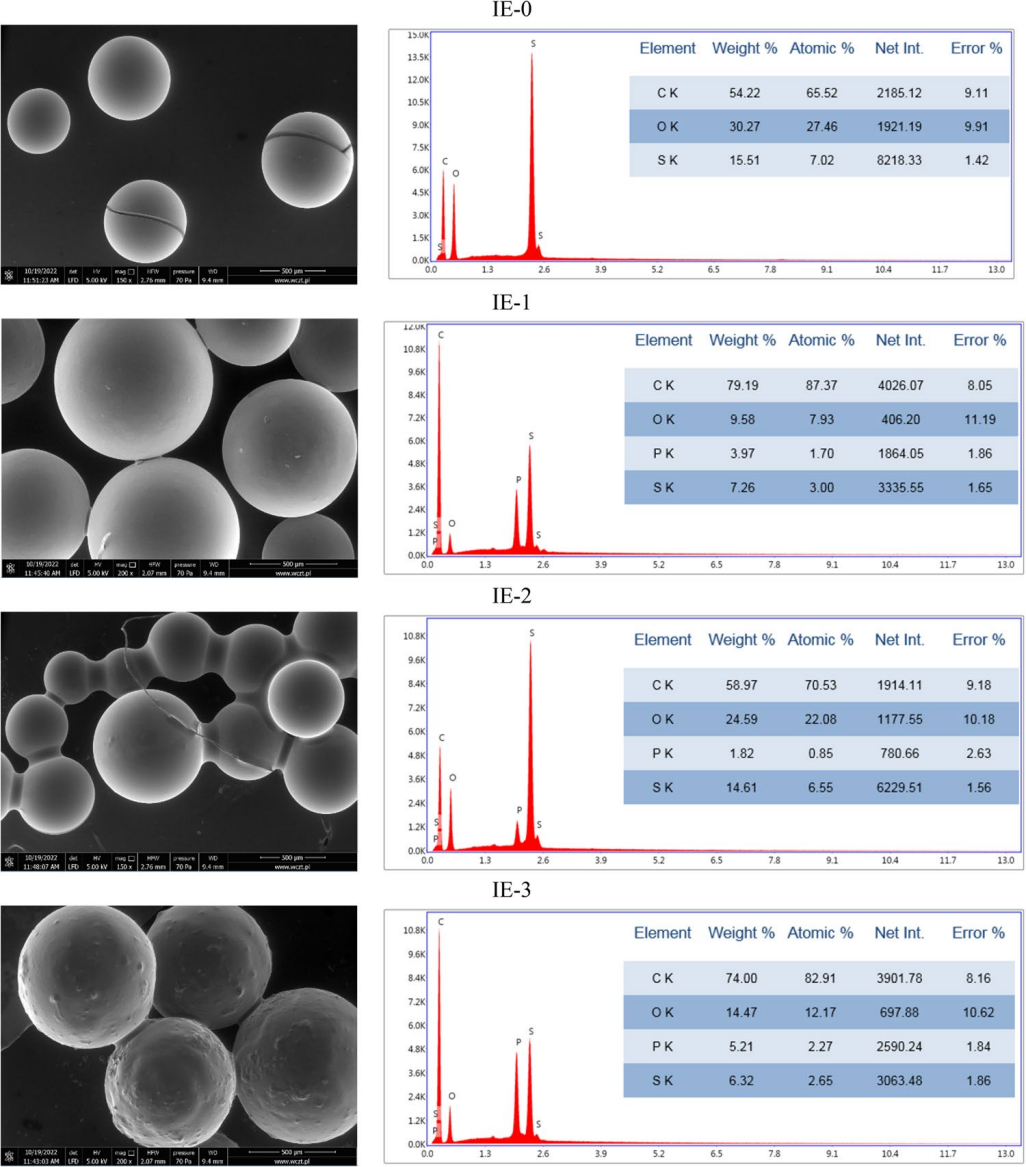
The SEM images of IEs before sorption together with EDS analysis

**Fig. 8:**
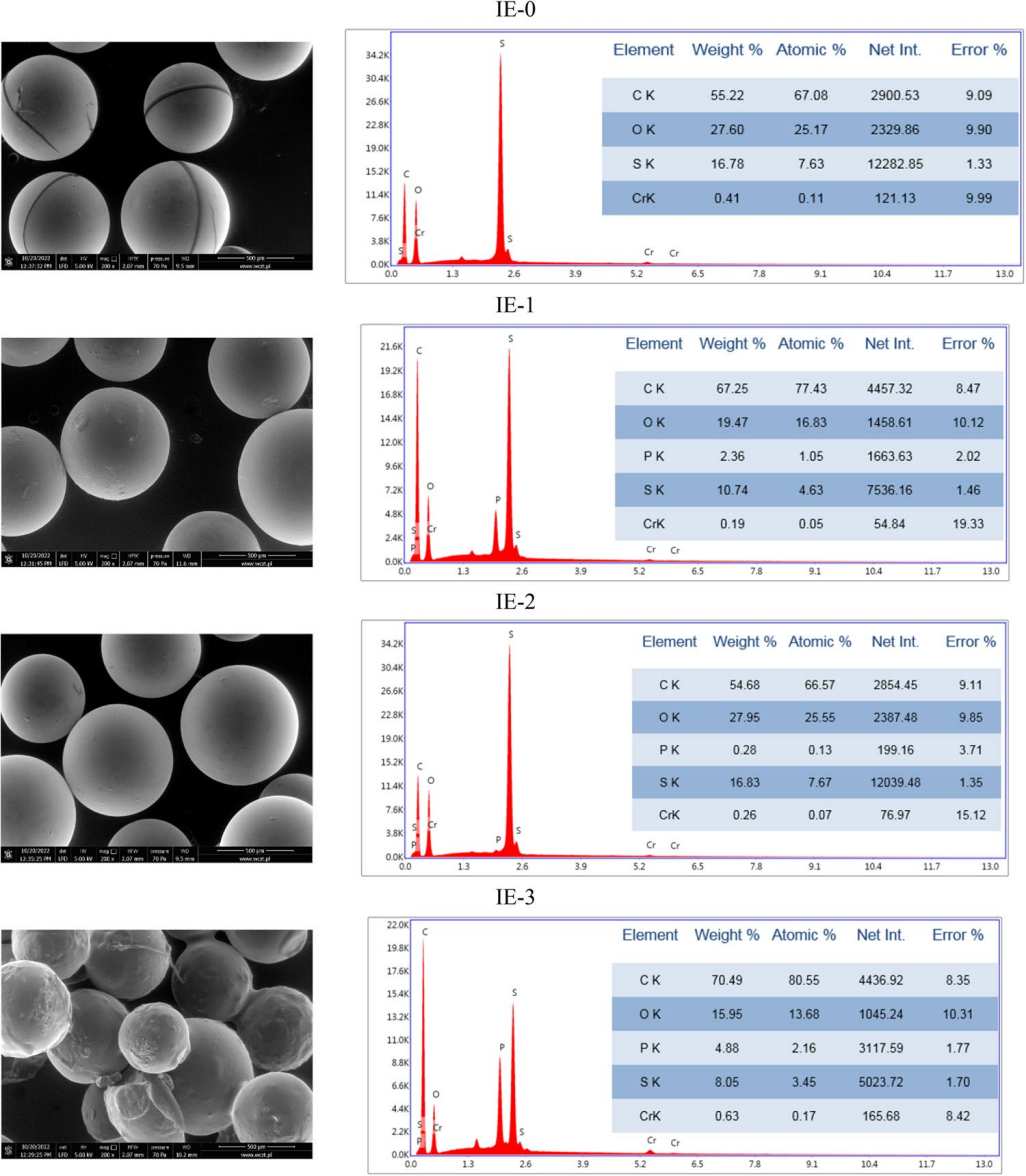
The SEM images of IEs after sorption together with EDS analysis

The grains of the tested ion exchangers are clearly visible (Fig. 7). The image of IE-0 shows a crack. The IE-1 grains are in a small degree glued together. In case of IE-2 these connections are much clearer. In turn, the grains in IE-3 have surface roughness. All these visible changes of the surfaces of IE-1, IE-2, and IE-3 in comparision with surface of IE-0 indicate the presence of the impregnation. The EDS analysis also confirms the presence of impregnates. In the graph of the IE-0, signs of C, O, and S are visible, in turn in the graphs of IE-1, IE-2, and IE-3 additional signs of P occurred. The carbon, oxygen and sulfur atoms come from used ion exchanger 001X8 H (together with functional groups R-SO_3_^−^), whereas the phosphorus present in all the investigated impregnates originate from the compounds used for impregnation (Cyphos IL 101, Cyphos IL 104, and D2EHPA, respectively). Hence, phosphorus is not visible in case of unimpregnated IE-0 Fig. 8.

Visible in the SEM images of IEs after sorption of chromium(VI) ions from aqueous solutions are slight changes in the images from the images obtained before sorption. The biggest changes are visible in the case of the grains shown in IE-2, whose surfaces are smooth and without junctions. That fact can indicate leaching of impregnate during the sorption. It probably had an influence on the sorption of chromium(VI) ions on this ion exchanger. The R_ads_ parameter in this case was very low and equaled 1.59%. The graphs of EDS analysis proved sorption of metal ions on the surfaces of the tested IEs, evidenced by the appearance of the signals of chromium atoms.

**Fig. 9:**
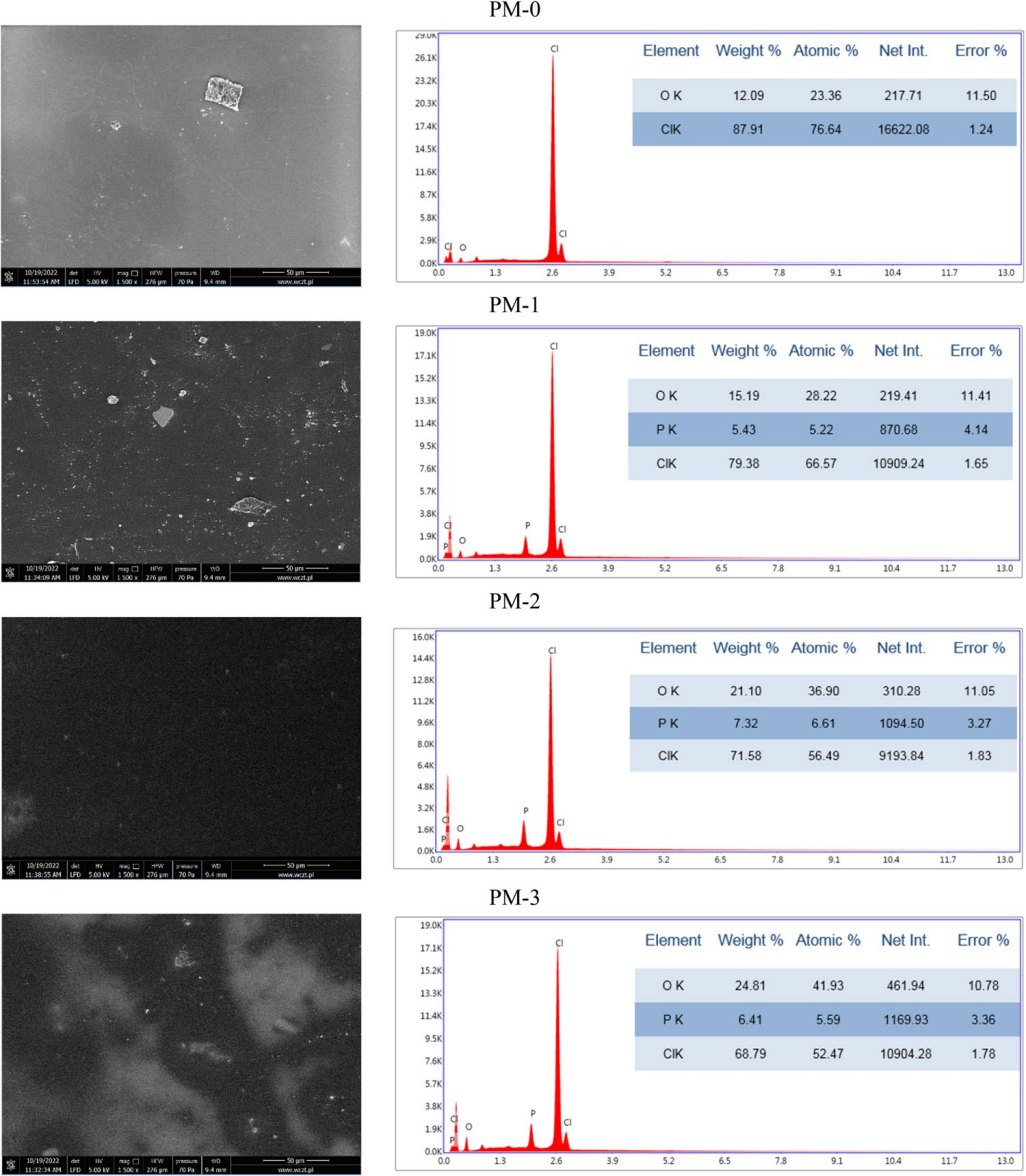
The SEM images of PMs before sorption together with EDS analysis

**Fig. 10:**
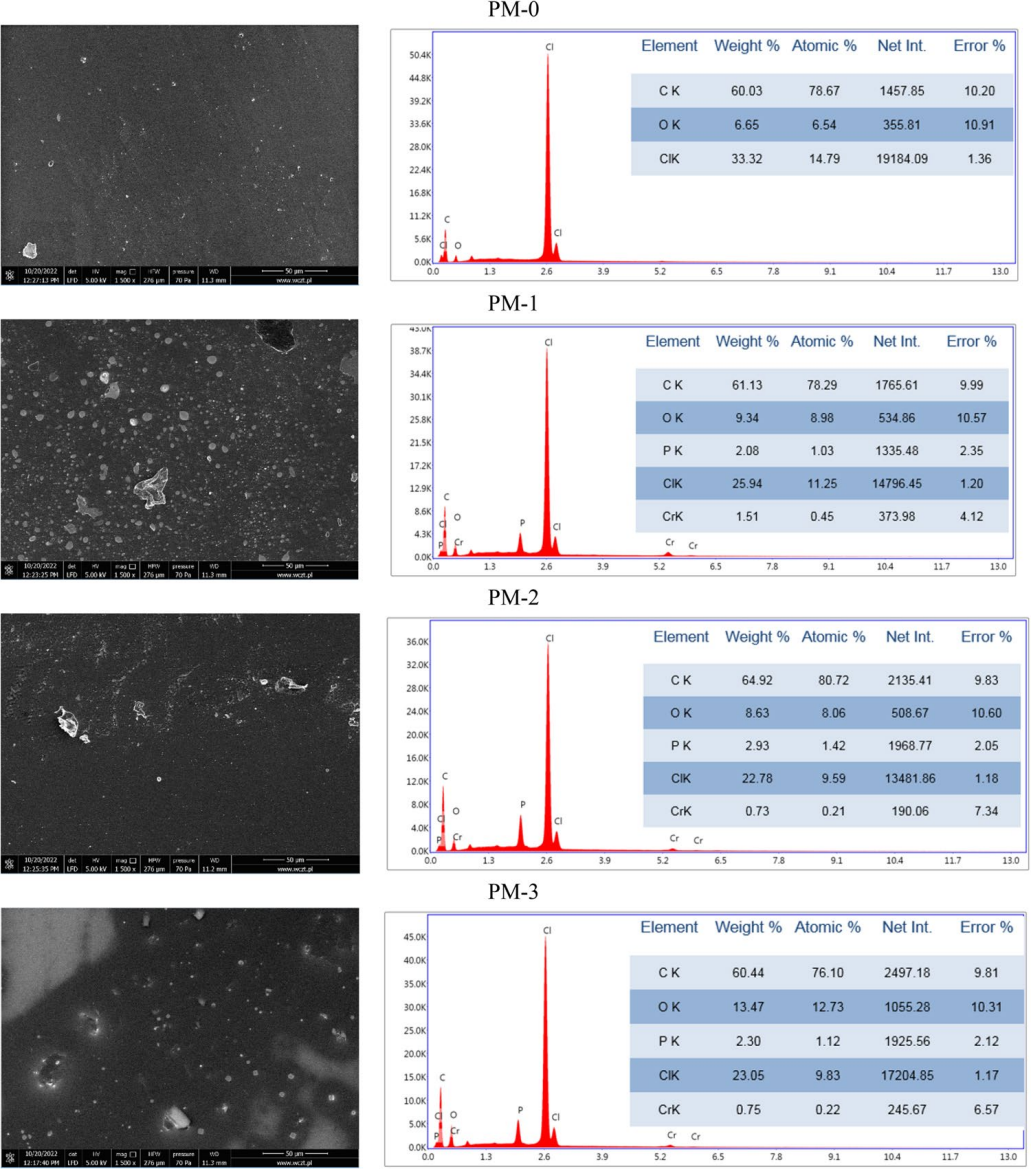
The SEM images of PMs after sorption together with EDS analysis

Much less information can be extracted from SEM images of polymer materials (Figs. 9 and 10). PMs contain lumps and this may indicate incomplete mixing of the ingredients. The EDS analysis proved that PMs were obtained of poly(vinyl chloride), which consists of C and Cl atoms. Impregnated PMs contain additional phosphorus atoms as do the previously mentioned impregnated IEs.

On the EDS graphs of PMs after sorption of metal ions (except PM-0) chromium atoms are visible. The absence of chromium on the graph of PM-0 confirmed the results obtained after sorption. The value of *R*_ads_ received on this polymer material was only 1.5%.

#### AFM

**Fig. 11:**
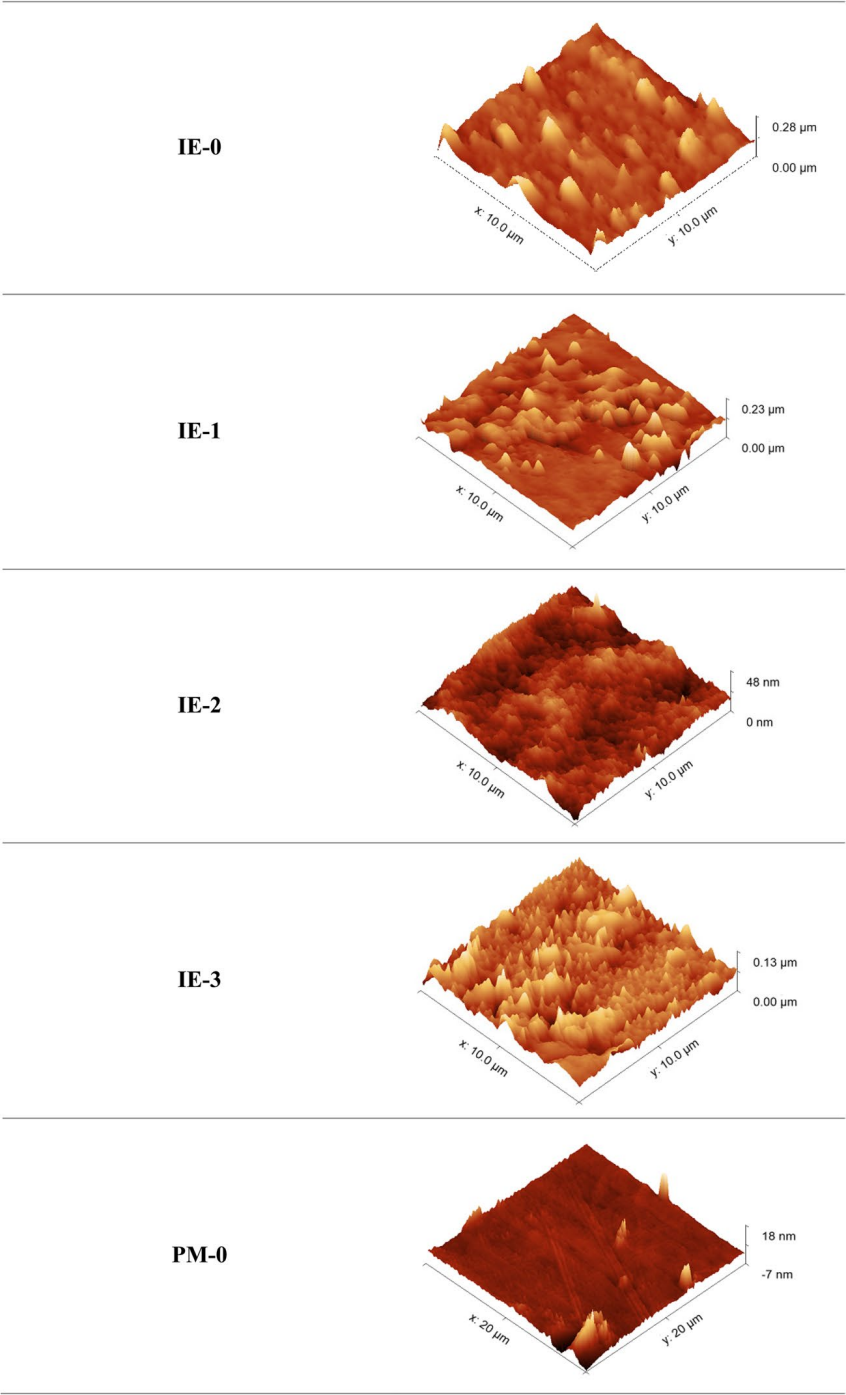

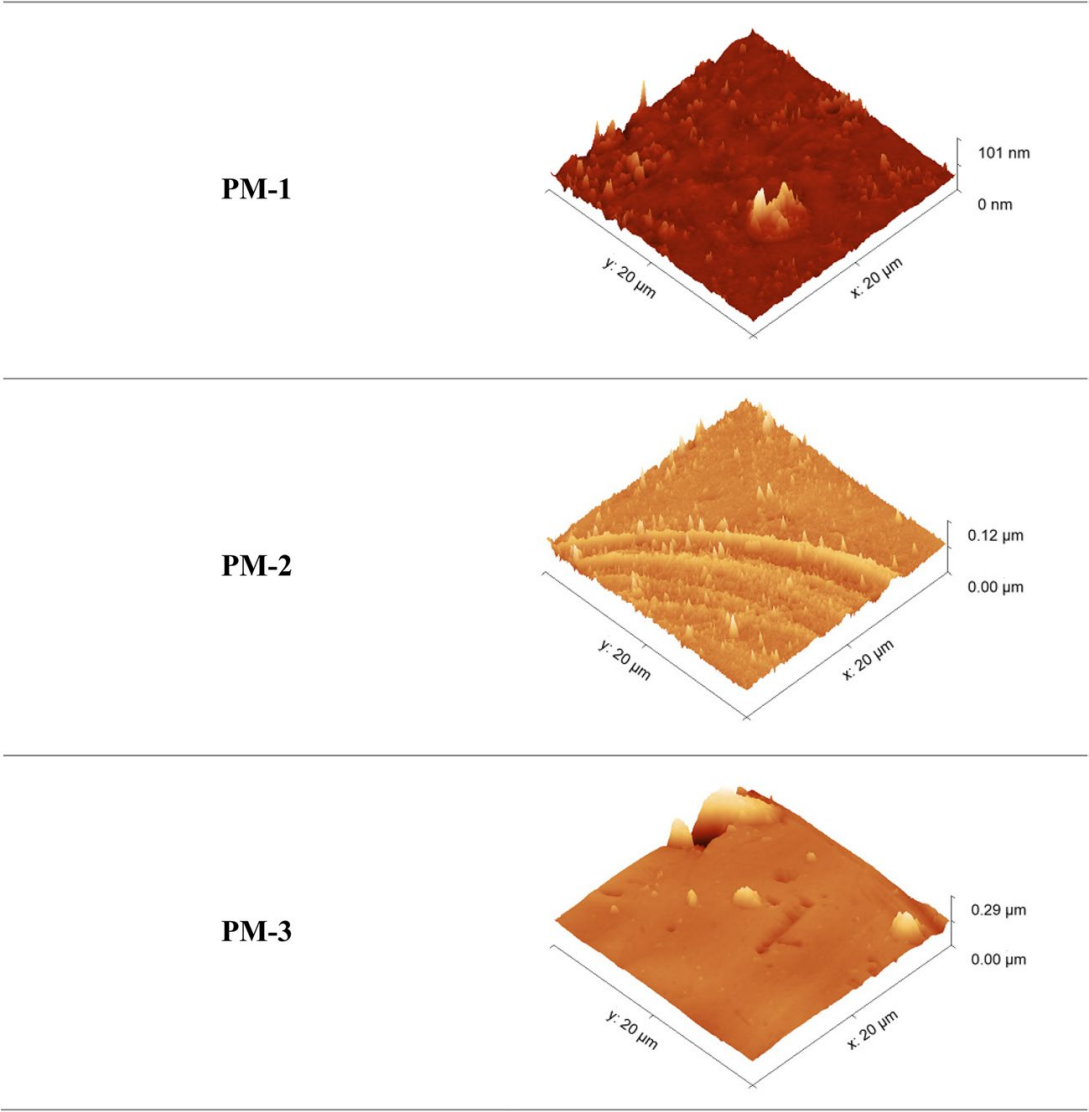
The 3D AFM images of sorption materials before sorption processes

Figure 11 shows AFM images of the tested sorption materials (ion exchangers: from IE-0 to IE-3, and polymer materials: from PM-0 to PM-3), in a three-dimensional form in the size of 10.0 × 10.0 μm. The AFM images have enabled to the assessment of the surface of formed materials.

The structure of all ion exchangers is characterized by roughness. Modification of IEs by carriers causes an increase in this roughness, and it is related to an increase in their active surface. The IE-3 has the most visible uneven surface. The same results were observed in the case of SEM analysis. In comparison to the IEs the surface of the tested polymer materials is more even. A visible inequality is shown by the PM-1 modified with Cyphos IL 101. The large active surface favors the sorption of metals on the surface of the sorbents, which is confirmed by the *R*_ads_% values obtained for IE-3 and PM-1 (Fig. 6).

#### FTIR-ATR

FTIR spectroscopy was used to detect the frequency variations of the functional groups in the investigated sorption materials (IEs and PMs) before and after sorption of chromium(VI) ions from aqueous solutions.

Figures S1–S16 (Supplementary materials) present in turn, the FTIR-ATR spectra of the investigated ion exchangers and polymer materials.

In all tested FTIR-ATR spectra the characteristic bands at 612 and 690 cm^–1^ and the bands from 2850 to 2960 cm^–1^ are visible, which correspond to the bending modes of the C-H bonds and to the -CH_2_- and -CH_3_ bonds, respectively.

The spectra of polymer materials (PMs) show the bands at 1254 and 1335 cm^–1^ corresponding to the stretching modes of the C−X bonds (where X is Cl) from poly(vinyl chloride). The band at 1426 cm^–1^, the bands from 2850 to 3000 cm^–1^ and from 3270 to 3330 cm^–1^ are also visible and correspond to the stretching modes of the C−H bonds. Moreover, the band at 1725 cm^–1^ can be attributed to the stretching mode of the C=O bond from the ADO molecule.

In turn, in the spectra of ion exchangers (IEs), the bands from 700 to 900 cm^–1^ and the bands from 1350 to 1450 cm^–1^ are shown, which can be attributed to S-OR and S=O bonds of functional groups of IE, respectively.

Finally, the spectra of IEs and PMs impregnated by Cyphos IL 101, Cyphos IL 104, and D2EHPA present bands from 950 to 1250 cm^–1^ corresponding to the P-H and P=O bonds.

The visibility of all characteristic bands in the FTIR-ATR-spectra confirmed the presence of individual components in the obtained sorbent material: a polymer–polystyrene (in IEs) or poly(vinyl chloride) (in PMs), a plasticizer—ADO and impregnate—Cyphos IL 101, Cyphos IL 104, or D2EHPA. It also suggested that there were no signs of the covalent bonds formation between the polymer, the plasticizer, and the impregnate. Only van der Waals or hydrogen bonds could be present.

The FTIR-ATR spectra of IEs and PMs before and after Cr(VI) ions sorption processes only show differences in the intensity of the characteristic bands.

## Comparison of performance of designed IEs and PMs with other sorption materials intended for Cr(VI) ions removal

Based on the conducted tests, it was found that the tested sorption materials (IEs and PMs) were successfully impregnated with ion carriers, and the use of some of them allowed for the effective removal of chromium(VI) ions from aqueous solutions. In the studies, the Cyphos IL 101, Cyphos IL 104, and D2EHPA were used, because they are well-known for the separation of metal ions via an ion-exchange mechanism, and thus the anionic species of metals are easily extracted by these compounds. In this study, the efficiency of removal of chromium(VI) ions using various sorption materials impregnated by ionic liquids (Cyphos IL 101 and Cyphos IL 104) and D2EHPA ranged from 1.59 to 82.51%. Some of the obtained sorption materials (i.e., IEs: IE-3; PMs: PM-1, PM-2) are competitive with respect to the most known sorbents recently used to bind chromium(VI) ions from aqueous solutions (Table 5).

**Table 5:** Comparison of the efficiency of sorption of chromium(IV) ions using various sorption materials

Sorption material	Efficiency of sorption	References
Chitosan fiber	42.8%	(Zhang et al. 2021)
Nanocomposite adsorbent GO-CS@MOF [Zn(BDC)(DMF)]	85%	(Samuel et al. 2018)
Chitosan-microcrystalline cellulose composite adsorbent	93%	(Yasmeen et al. 2016)
Chitosan-based hydrogel	94.72%	(Vilela et al. 2019)
Serpentine	81.66%	(Shaban et al. 2018)
Composite adsorbent (CCN-PEI)	65%	(Liu et al. 2017)
Nanocomposite adsorbent (Fe_3_O_4_@Arg-PPy)	64%	(Chigondo et al. 2019)

Results reported by Zhang et al. (2021) show that utilization of chitosan fiber as a green material allowed for removal of 42.8% of chromium(VI) ions. A novel nanocomposite adsorbent material (Samuel et al. 2018) has been synthesized using chitosan (CS), graphene oxide (GO), and metal-organic framework (MOF[Zn(BDC)(DMF)]) and used for the adsorption of chromium(VI). It was reported that with addition of K_2_Cr_2_O_7_ into the real wastewater, the GO-CS@MOF [Zn(BDC)(DMF)] composite material allowed obtaining of sorption capacity of 85%. In addition, it was found that application of synthesized bio-adsorbent chitosan-microcrystalline from waste materials of shrimp processing industries and waste cotton rags of garments industries enables for removal of 93% of chromium(VI) ions from tannery effluent (Yasmeen et al. 2016). Vilela et al. (2019) studied the adsorption of chromium(VI) ions in aqueous solutions using a chitosan-based hydrogel synthesized from radical chitosan, polyacrylic acid, and *N,N*′-methylenebisacrylamide. The percentage removal of chromium(VI) ions at pH 4.5 and 100 mg/L initial metal concentration was 94.72%. Also, natural mineral layered magnesium silicate (serpentine) can be an effective adsorbent to remove Cr(VI) ions from water pollutants. The best results of removal of Cr(VI) ions (81.66%) was obtained using a 25 mg/L initial concentration (Shaban et al. 2018). Liu et al. (2017) reported that using a composite adsorbent (CCN-PEI) composed of carboxylated cellulose nanocrystals (CCN) and polyethyleneimine (PEI) allowed for removal of 65% of chromium(VI) ions within the first 100 min. of the process. They found that the adsorption equilibrium was reached within 250 min. Chigondo et al. (2019) fabricated an arginine-functionalized polypyrrole nanocomposite (Fe_3_O_4_@Arg-PPy) for the removal of toxic chromium(VI) ions. They reported that the adsorbent could be regenerated for four adsorption–desorption cycles (adsorption efficiency was 64%). Such a large variety of materials designed and used to remove hexavalent chromium ions from aqueous solutions is due, inter alia, to the fact that even if a specific method allows for the removal of this toxic contamination with good efficiency, it usually has serious limitations (e.g., the time-consuming process of preparing the sorption material (Yasmeen et al. 2016). In the case of the sorption materials we prepared and applied, such as polymer materials PM-1, PM-2, and ion exchanger IE-3, not only is the efficiency of the Cr(VI) ions sorption process high (respectively: PM-1 82.51%, PM-2 73.91%, IE-3 75.08%), but the formulation of sorption materials is relatively simple and not time-consuming, and all the needed chemicals are well-known (there is no risk that they pose a threat to the environment in the amounts used) and commercially available.

## Conclusions

The results of the application of D2EHPA and ionic liquids Cyphos IL 101 and Cyphos IL 104 as active compounds in developed polymer materials and ion exchangers intended for the removal of hexavalent chromium ions from aqueous model solutions show that the effectiveness of these well-known compounds varies depending on the type of material in which they are used. When the abovementioned compounds were applied in polymer materials, the efficiency of the binding of chromium(VI) ions from aqueous solutions by the obtained PMs was as follows: PM-1 with Cyphos IL 101 (82.51%) > PM-2 containing Cyphos IL 104 (73.91% ) > PM-3 with D2EHPA (35.69%) > PM-0 containing only polymer and plasticiser (1.50%). In the case of ion exchangers impregnated with the analyzed compounds, the effectiveness of the method decreased as follows: IE-3 with D2EHPA (75.08%) > IE-1 containing Cyphos IL 101 (40.61%) > IE-2 with Cyphos IL 104 (1.59%) > IE-0 with no active compound (0.27%). The results of the desorption experiments carried out for all tested PMs and IEs show that almost all chromium(VI) ions bound on the formulated sorption materials can be transferred to the acidic solutions. The results of chromium(VI) ions desorption for polymer materials were: 75.85% for PM-1, 68.70% for PM-2, 33.21% for PM-3 and 0.00% in the case of PM-0, while for ion exchangers: 72.99% for IE-3, 40.61% for IE-1 and 0.00% for IE-2 and IE-0. Interestingly, D2EHPA, the use of which in polymer materials led to the worst sorption results of hexavalent chromium ions, performed best as an active compound in the ion exchanger. On the other hand, Cyphos IL 101 and 104 allowed for effective elimination of Cr(VI) ions, being active compounds of polymer materials, but they performed much worse as impregnates of ion exchangers. These results clearly indicate that not only the selection of the appropriate active compounds for binding toxic chromium(VI) ions plays a role, but the choice of proper separation method in which such compounds will be used is extremely important. This is also confirmed by the results of the solvent extraction, in which the best results regarding the elimination of hexavalent chromium ions were obtained using Cyphos IL 104 (64.15%—Cyphos IL 104, 46.72%—Cyphos IL 101, 5.84%—D2EHPA). Additionally, application in this study of methods, such as FTIR-ATR, SEM-EDS, and AFM allowed for the characterization of the surfaces of the formulated impregnated ion exchangers and polymer materials and for the confirmation of the sorption of chromium(VI) ions from aqueous solutions on developed sorption materials. On the bases of the obtained results, it can be concluded that methods based on the use of polymer materials containing Cyphos IL 101 and Cyphos IL 104 and the ion exchanger with D2EHPA have a particularly high potential for removing hexavalent chromium ions, which are dangerous environmental contaminants. Both the polymer materials and the ion exchangers, the formulation of which is related to utilization of small amounts of active chemical compounds and low consumption of toxic solvents, are environmentally friendly methods that additionally afford the possibility of multiple use of the material after regeneration/desorption. From an economic point of view, it is also significant that the formulation of polymer materials and ion exchangers is not only a relatively fast but also a simple process that requires neither complicated operations nor complex devices.

### Supplementary Information

ESM 1

## Data Availability

Not applicable.
