# Supplementary material for: Efficient, fast, simple, and eco-friendly methods for separation of toxic chromium(VI) ions based on ion exchangers and polymer materials impregnated with Cyphos IL 101, Cyphos IL 104, or D2EHPA
Source: Environ Sci Pollut Res Int. 2024 Jan 4;31(5):7977–93. doi: 10.1007/s11356-023-31648-5 (PMC10821846; doi:10.1007/s11356-023-31648-5)
Supplement: Supplementary file 1 — ESM 1 [file 11356_2023_31648_MOESM1_ESM.doc]

Environmental Science and Pollution Research

Supplementary materials to the manuscript:

**Efficient, fast, simple and eco-friendly methods for separation of toxic chromium(VI) ions based on ion exchangers and polymer materials impregnated with Cyphos IL 101, Cyphos IL 104 or D2EHPA**

**Katarzyna Witt^1*^, Małgorzata A. Kaczorowska^1^, Daria Bożejewicz^1^**

Faculty of Chemical Technology and Engineering, Bydgoszcz University of Science and Technology, 3 Seminaryjna Street, PL 85326 Bydgoszcz, Poland

**^*^**Corresponding author: e-mail: Katarzyna.Witt@pbs.edu.pl; phone: +48 52 3749055

The figures below present the FTIR-ATR spectra of the investigated ion exchangers IEs and polymeric materials PMs.


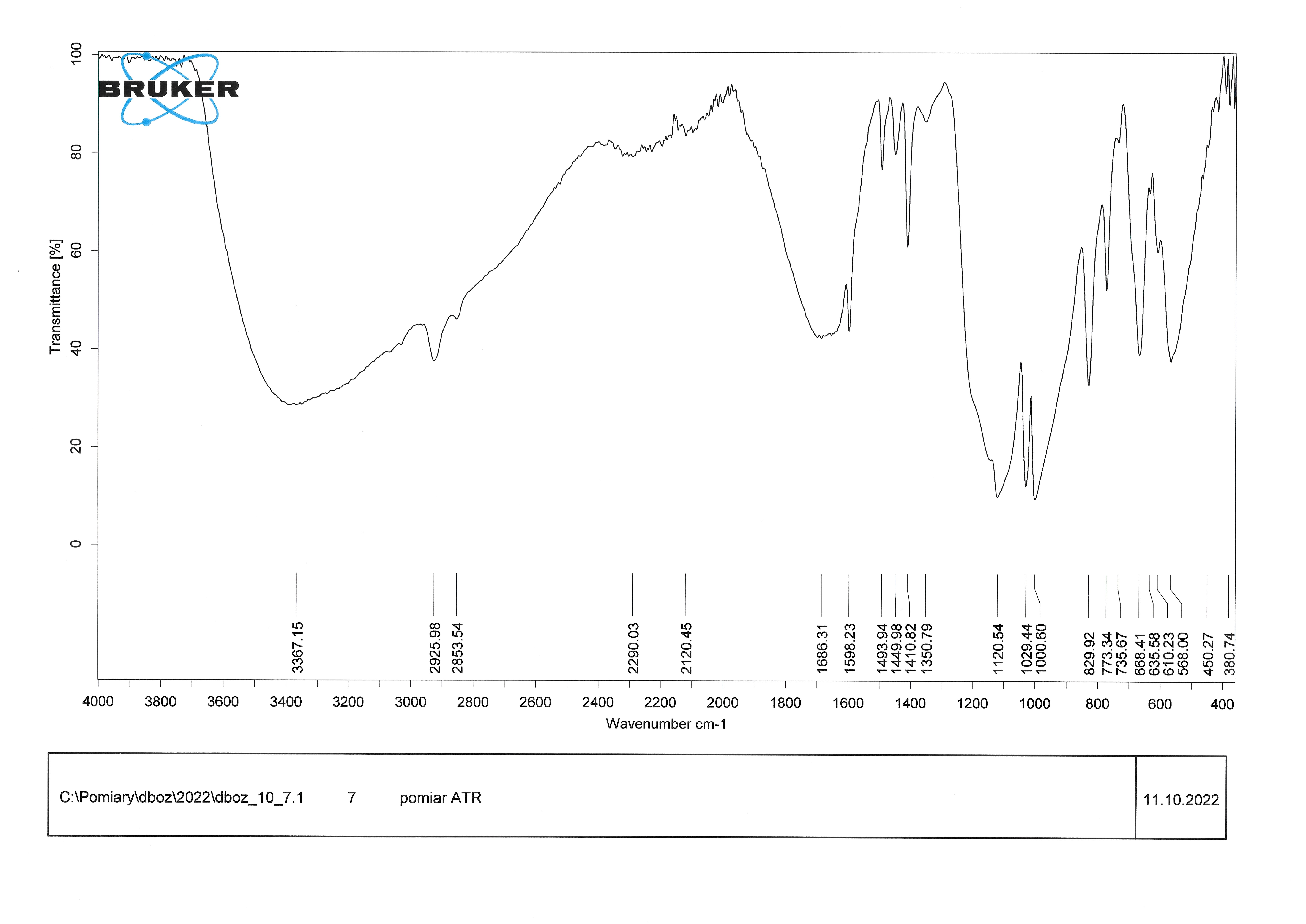


Figure S1. FTIR – ATR spectrum of IE-0 before sorption of Cr(VI) ions.


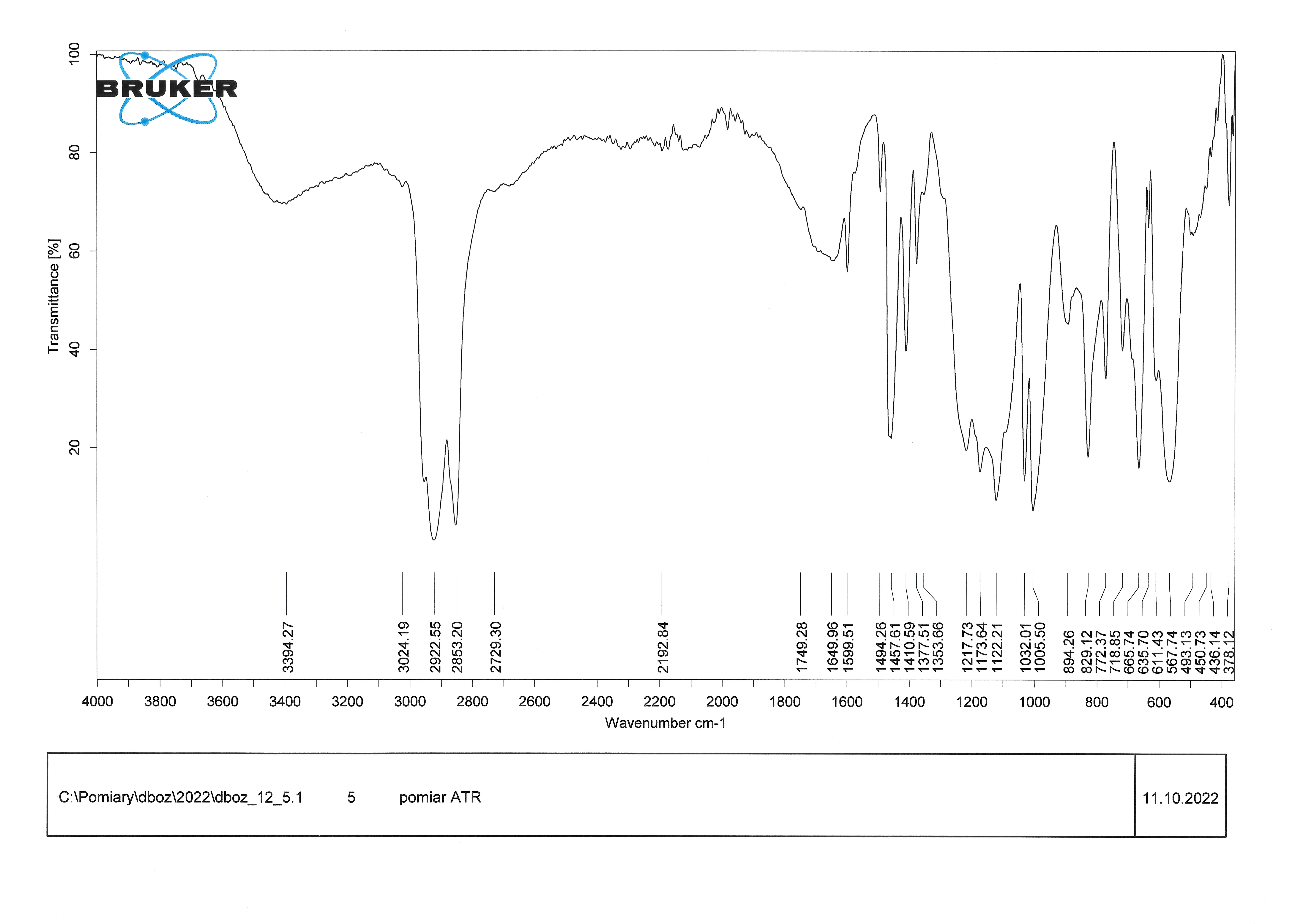


Figure S2. FTIR – ATR spectrum of IE-1 before sorption of Cr(VI) ions.


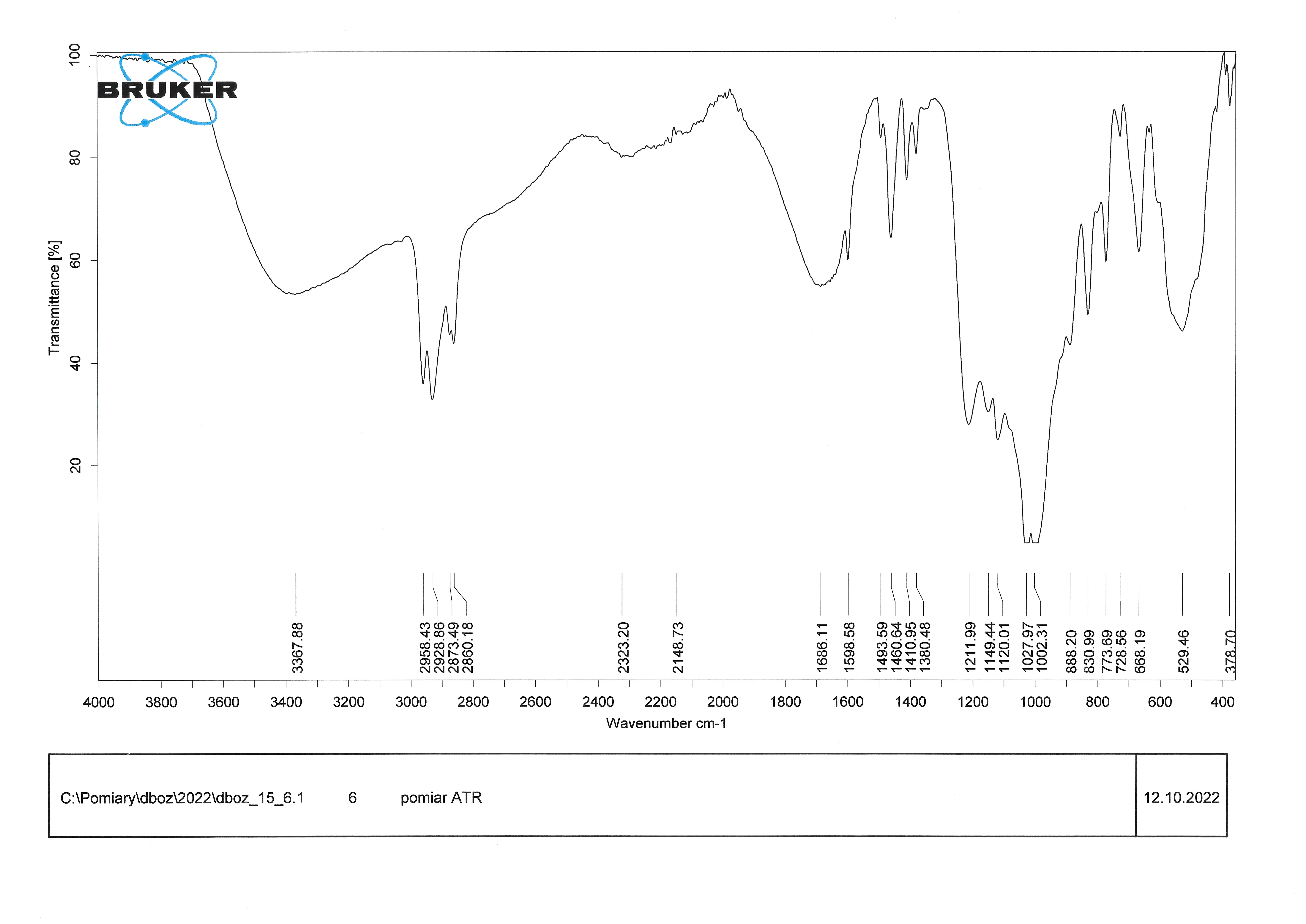


Figure S3. FTIR – ATR spectrum of IE-2 before sorption of Cr(VI) ions.


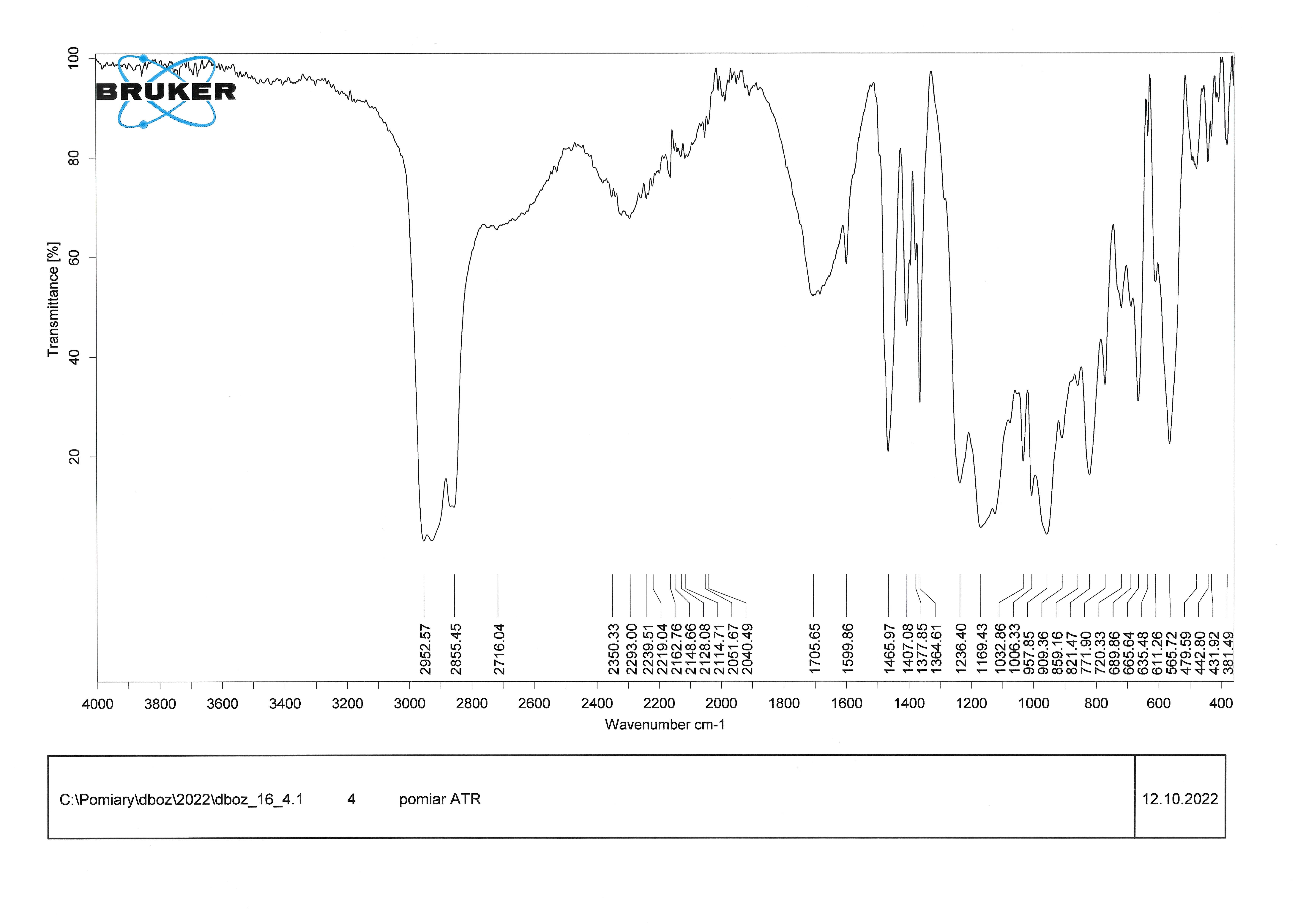


Figure S4. FTIR – ATR spectrum of IE-3 before sorption of Cr(VI) ions.


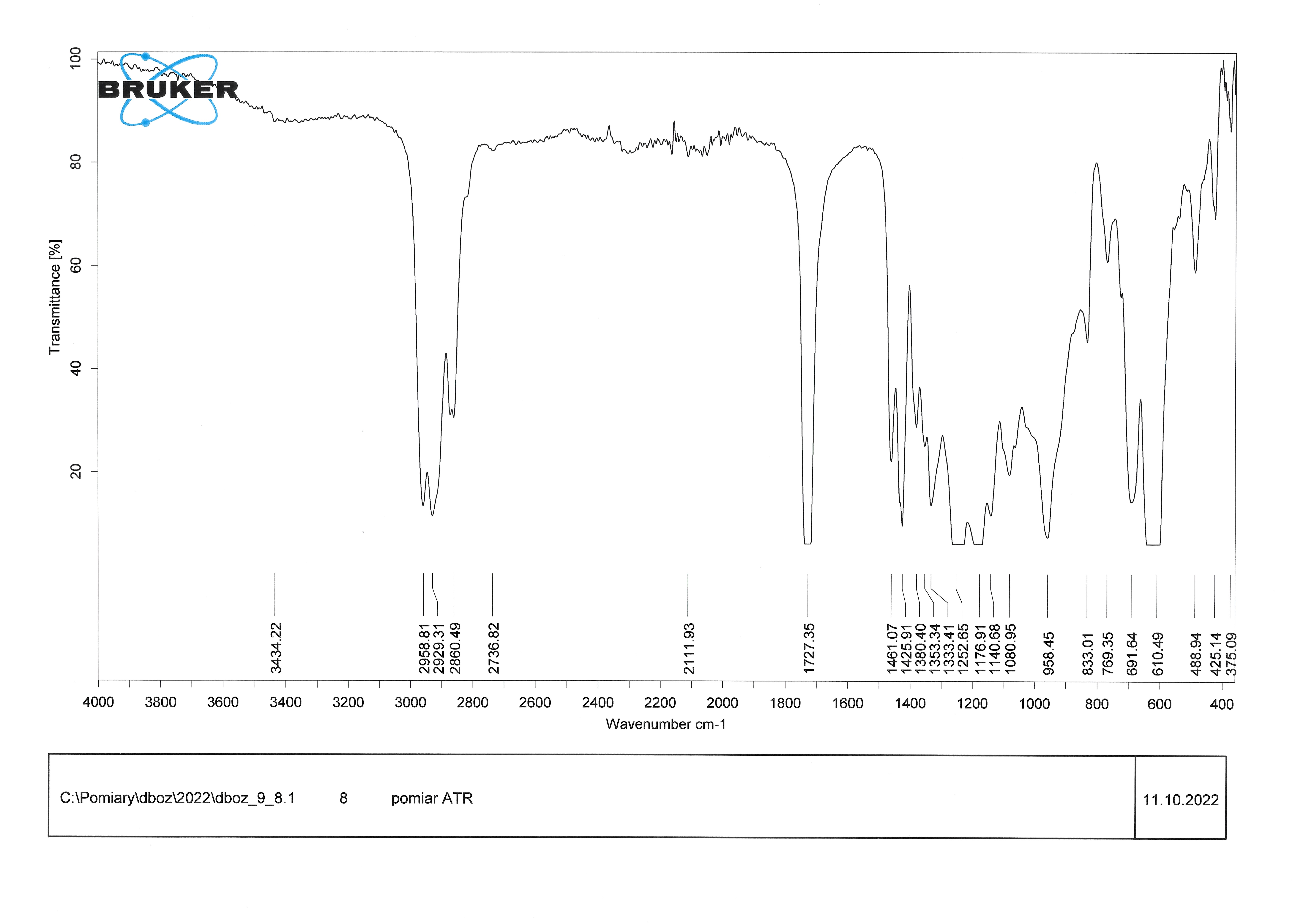


Figure S5. FTIR – ATR spectrum of PM-0 before sorption of Cr(VI) ions.


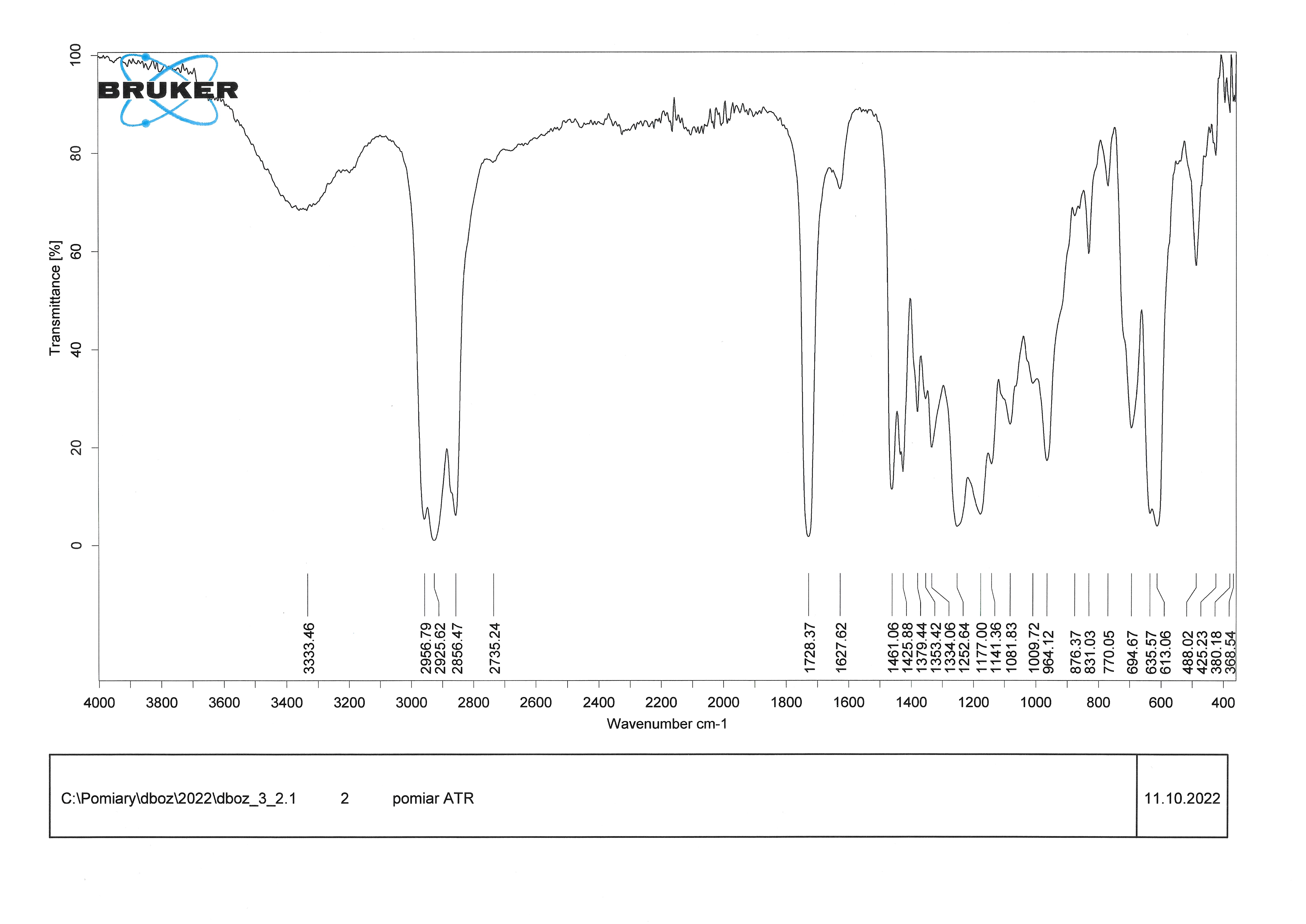


Figure S6. FTIR – ATR spectrum of PM-1 before sorption of Cr(VI) ions.


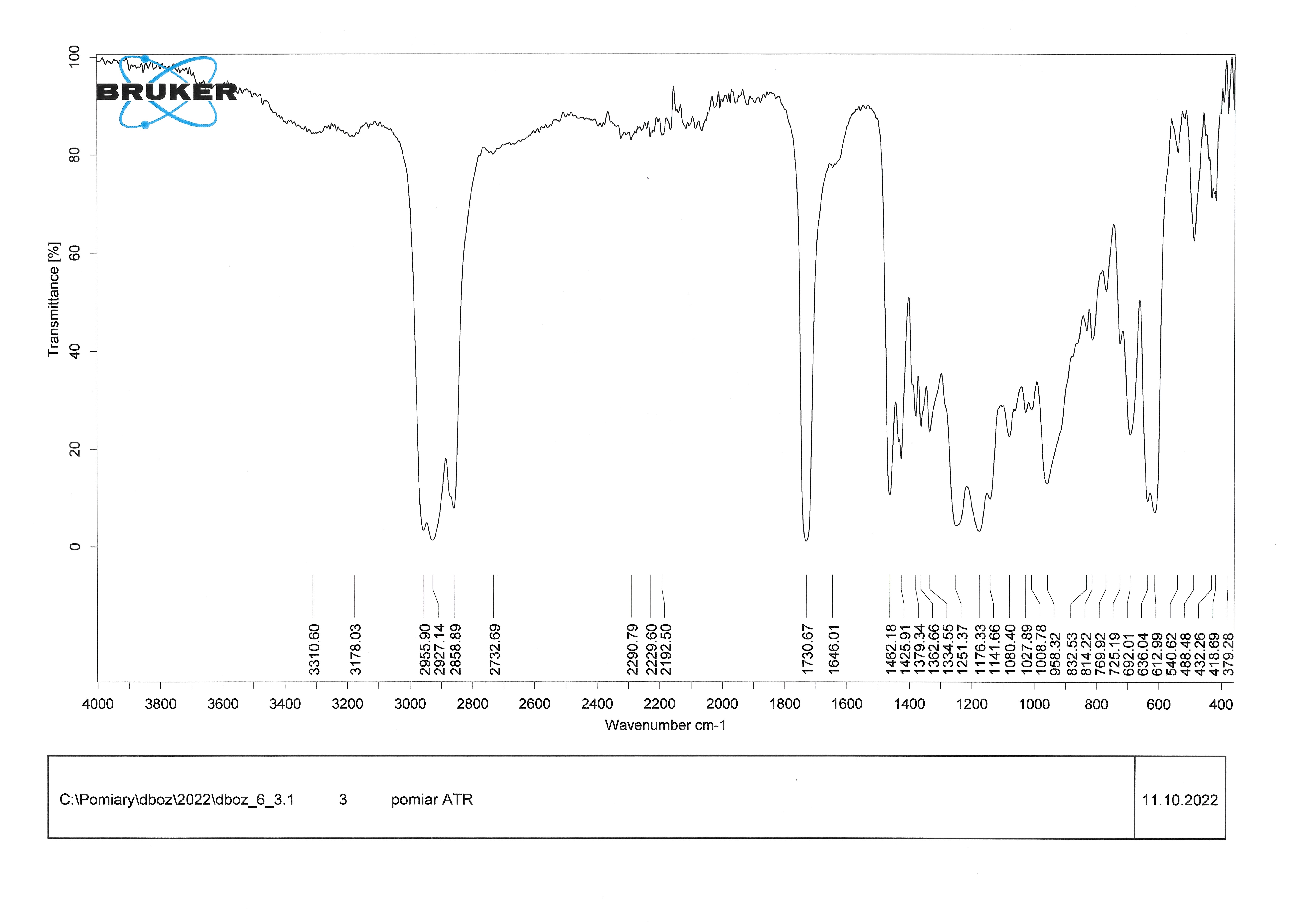


Figure S7. FTIR – ATR spectrum of PM-2 before sorption of Cr(VI) ions.


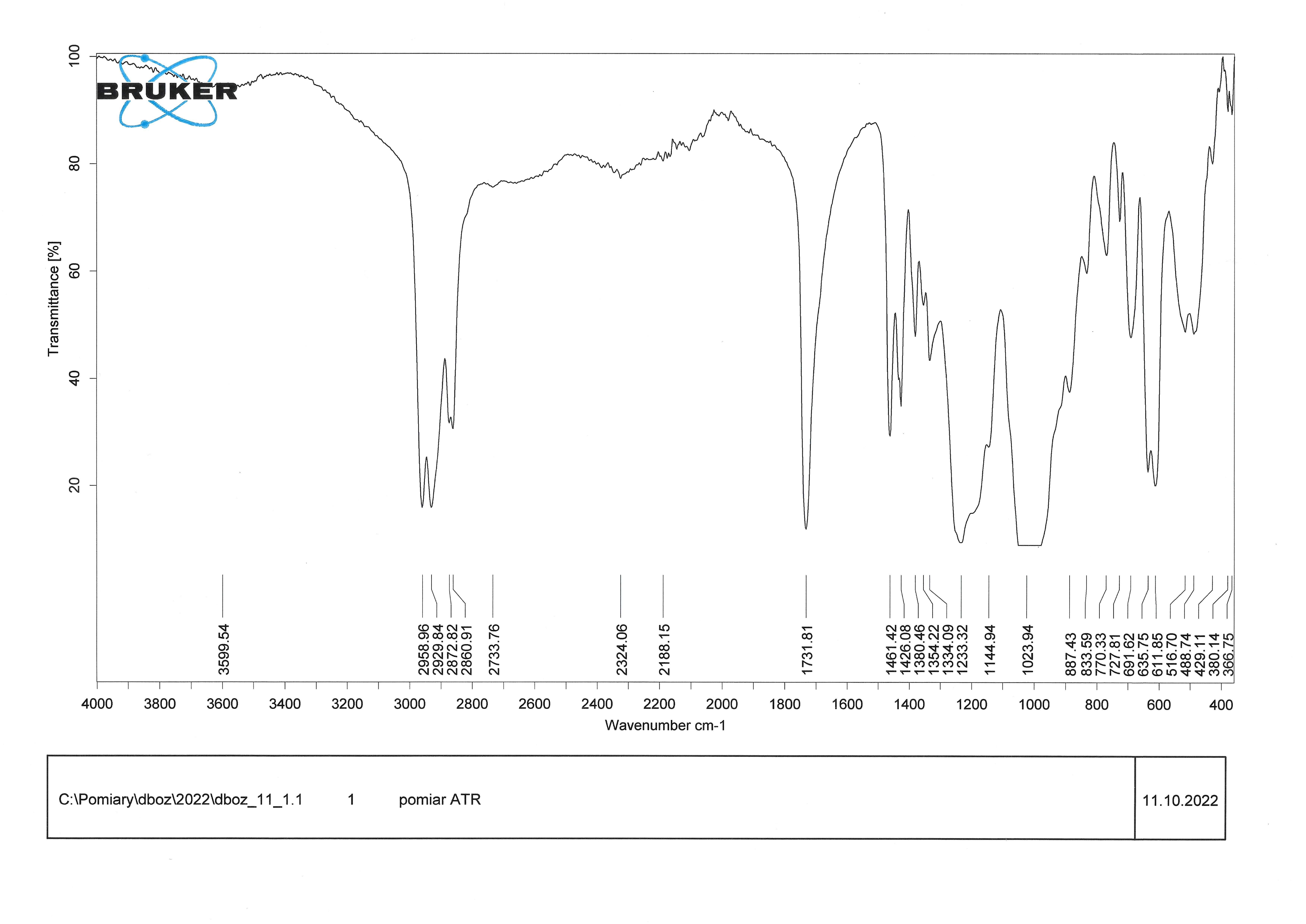


Figure S 8. FTIR – ATR spectrum of PM-3 before sorption of Cr(VI) ions.


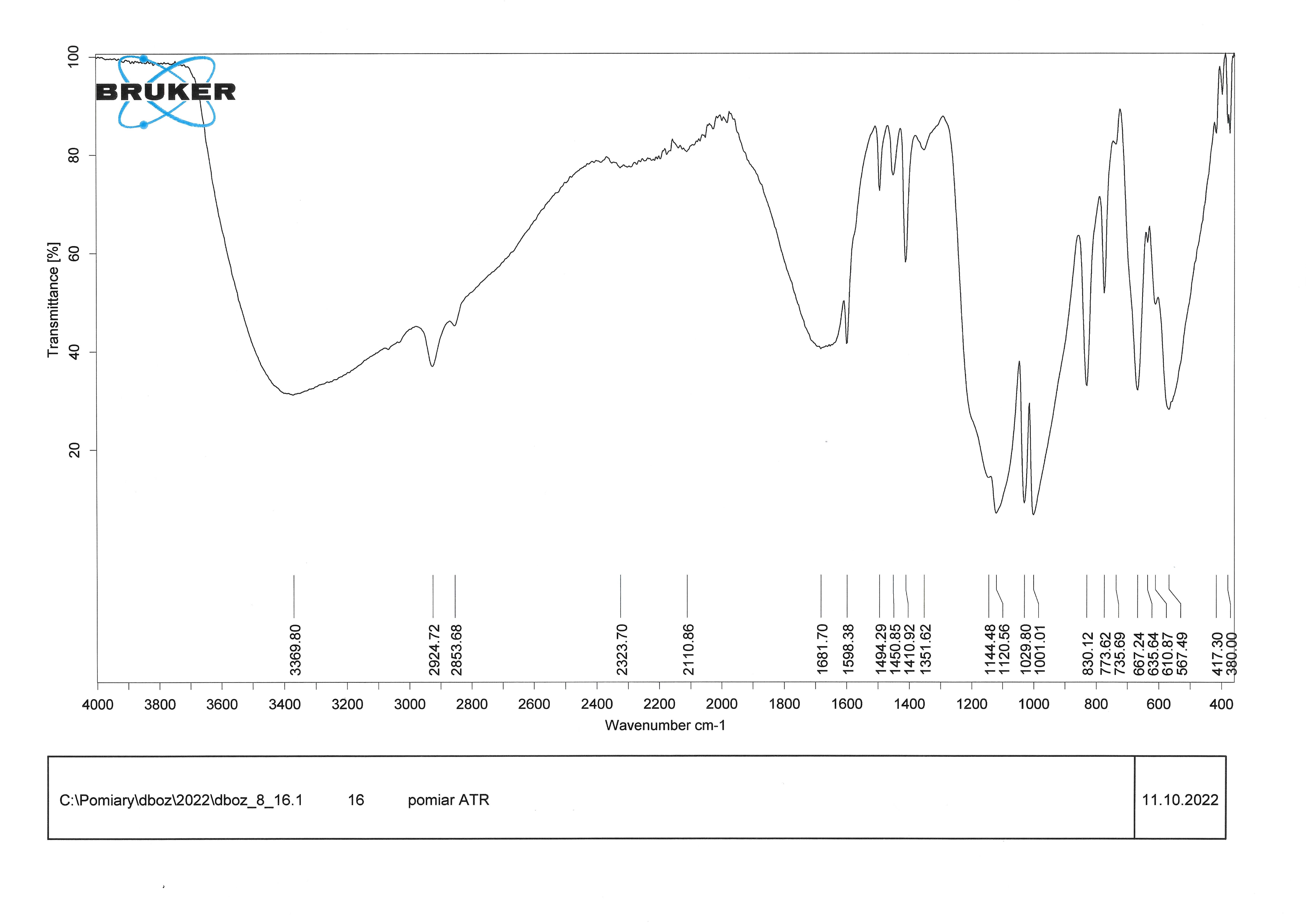


Figure S9. FTIR – ATR spectrum of IE-0 after sorption of Cr(VI) ions from aqueous solution.


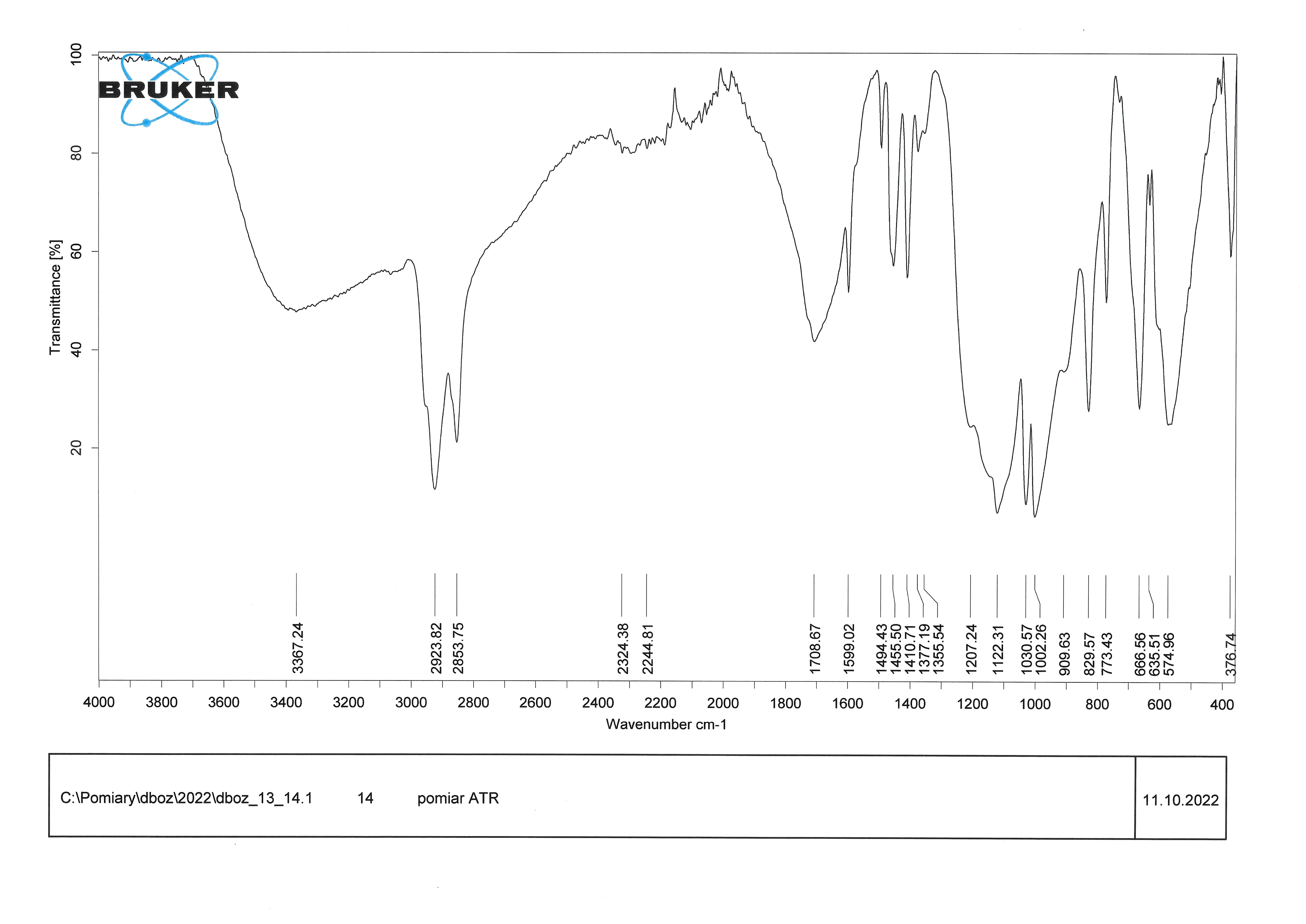


Figure S10. FTIR – ATR spectrum of IE-1 after sorption of Cr(VI) ions from aqueous solution.


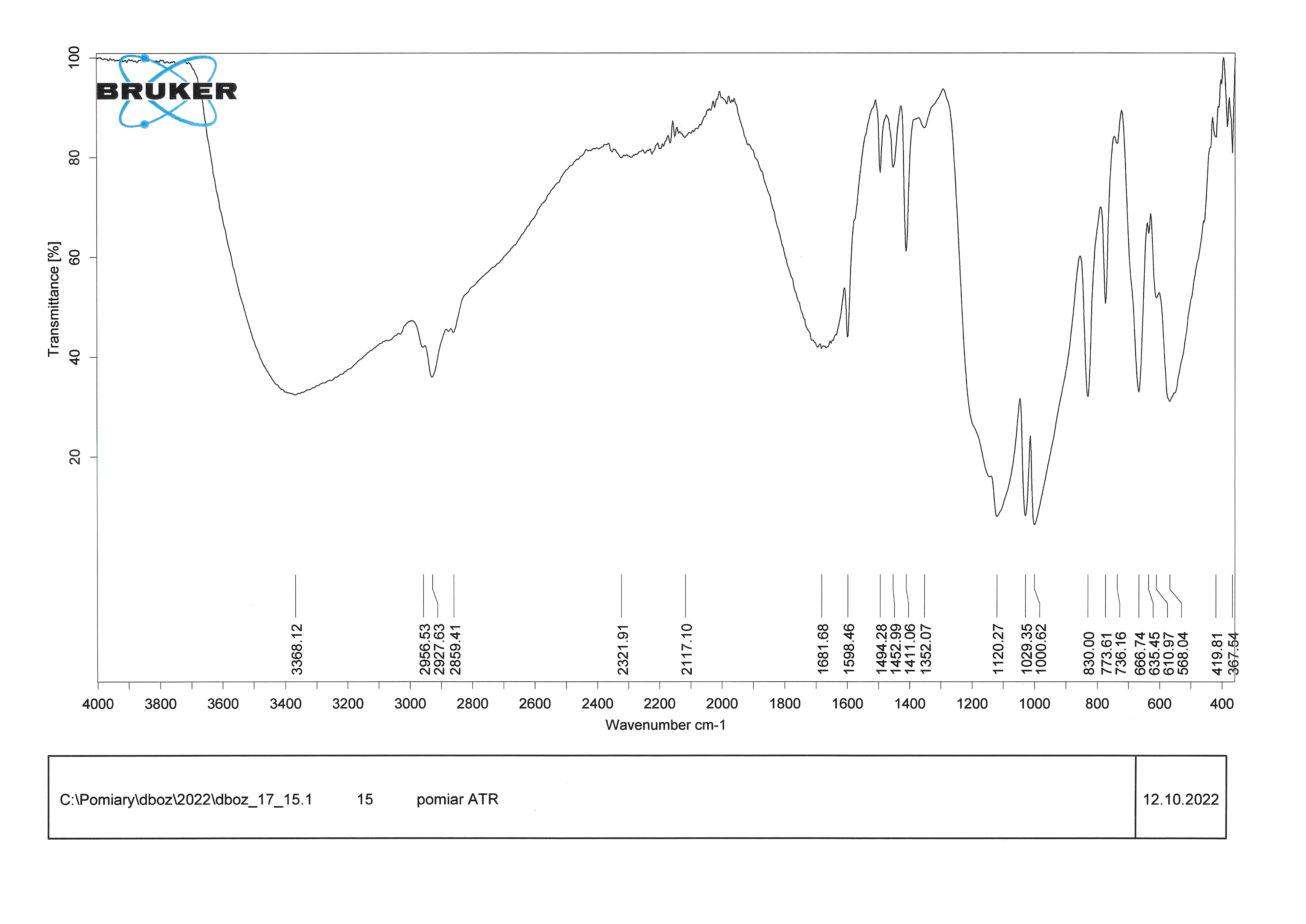


Figure S11. FTIR – ATR spectrum of IE-2 after sorption of Cr(VI) ions from aqueous solution.


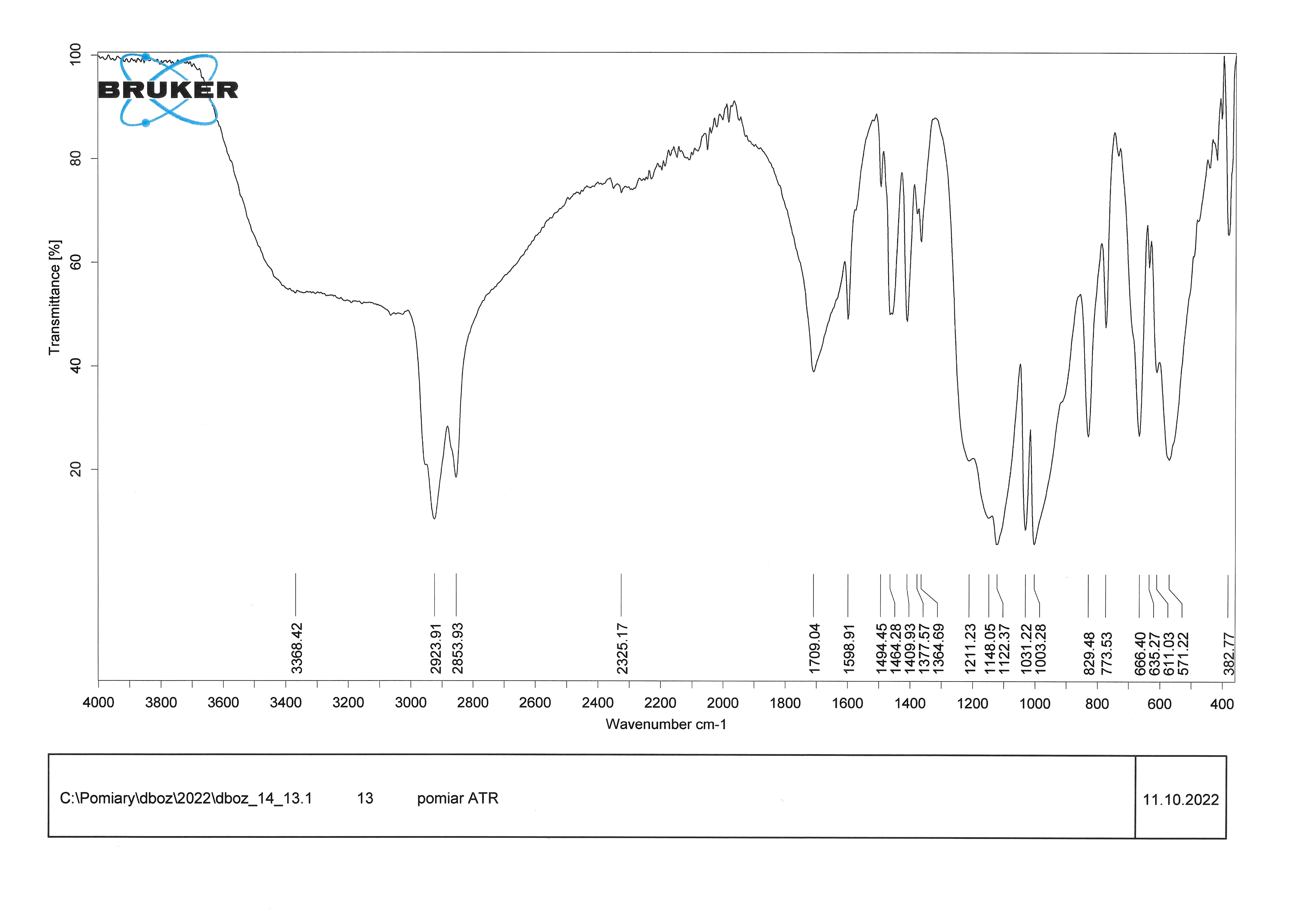


Figure S12. FTIR – ATR spectrum of IE-3 after sorption of Cr(VI) ions from aqueous solution.


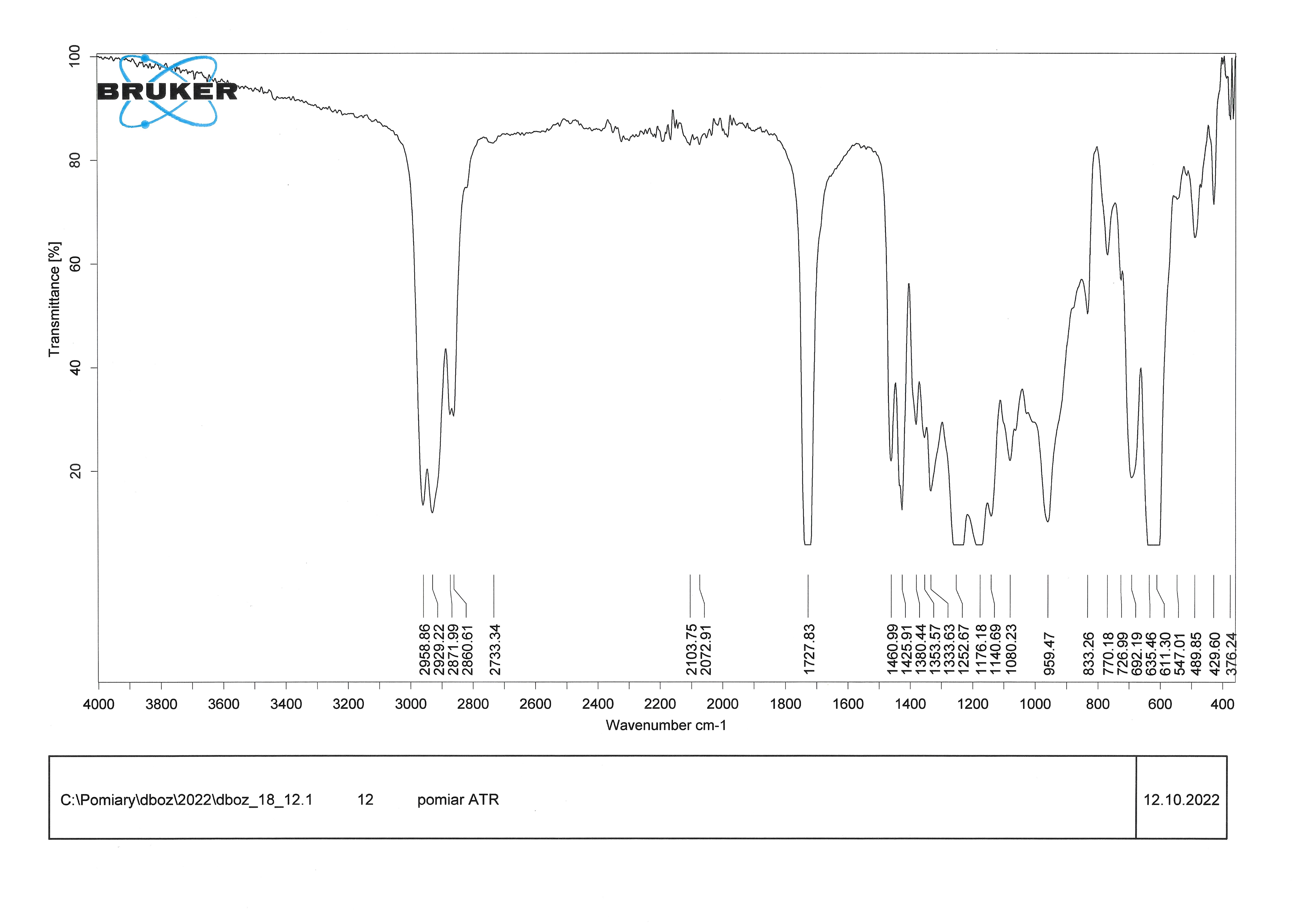


Figure S13. FTIR – ATR spectrum of PM-0 after sorption of Cr(VI) ions from aqueous solution.


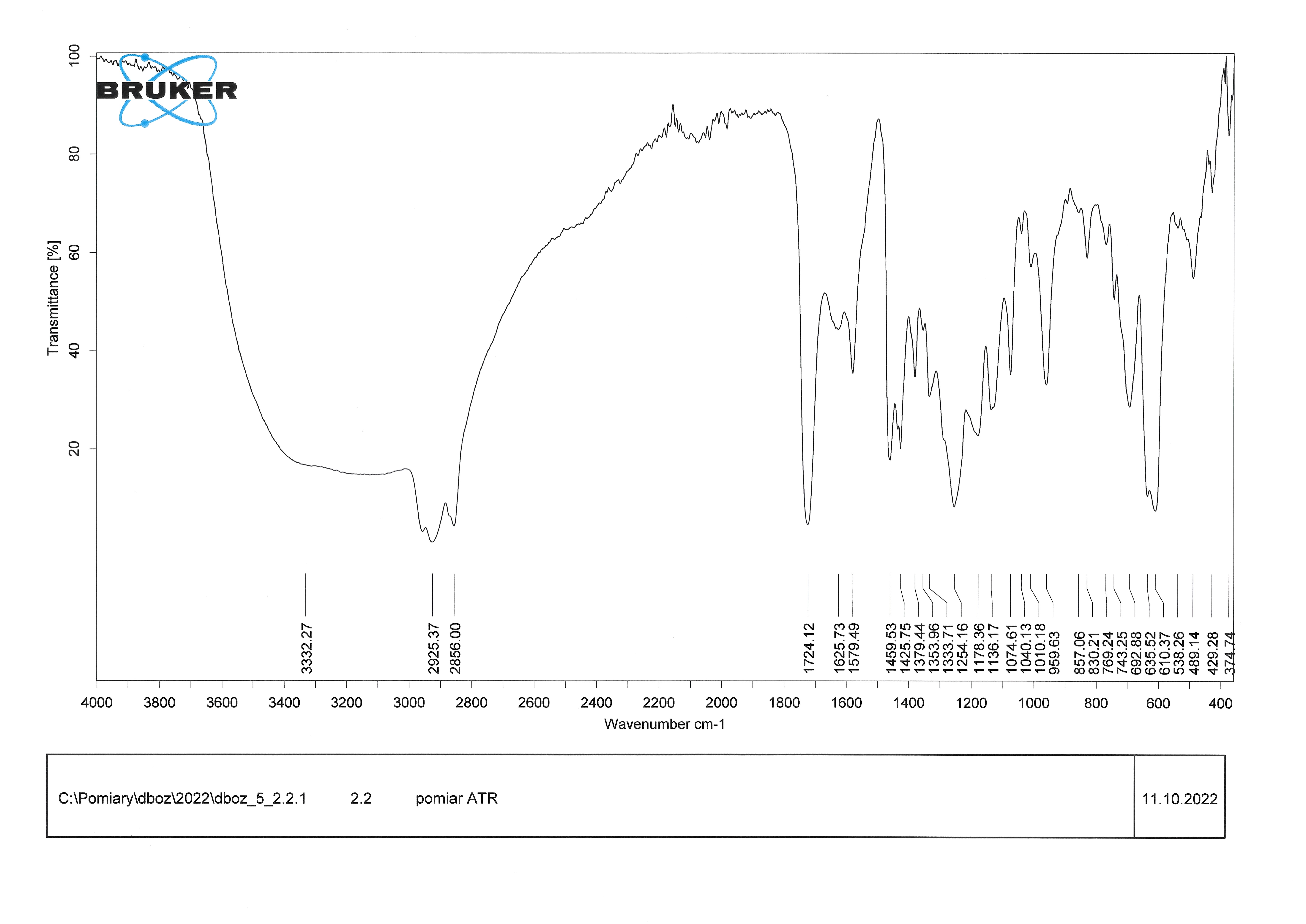


Figure S14. FTIR – ATR spectrum of PM-1 after sorption of Cr(VI) ions from aqueous solution.


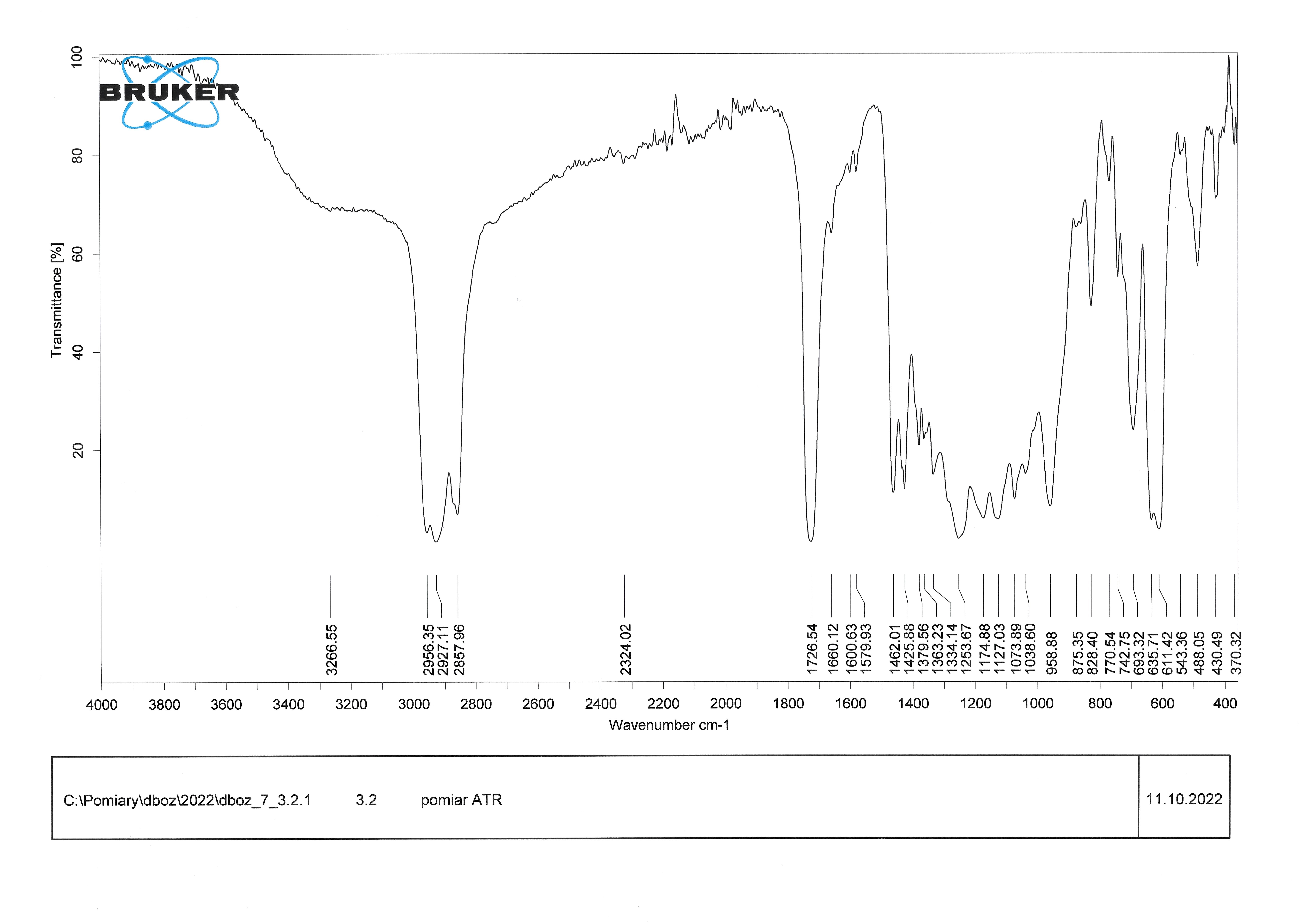


Figure S15. FTIR – ATR spectrum of PM-2 after sorption of Cr(VI) ions from aqueous solution.


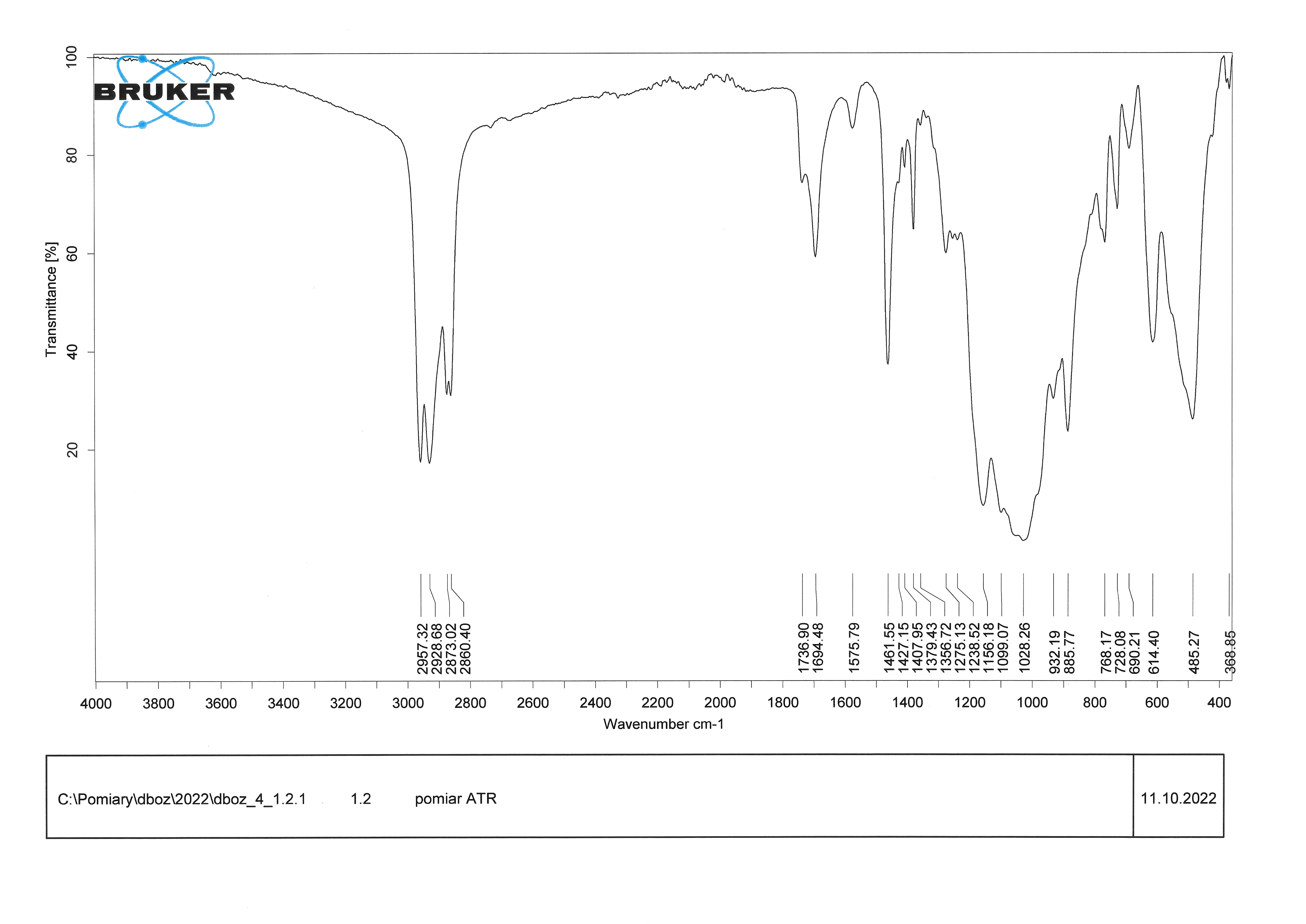


Figure S16. FTIR – ATR spectrum of PM-3 after sorption of Cr(VI) ions from aqueous solution.
